# Exploitation of Black Olive (*Olea europaea* L. cv. Piantone di Mogliano) Pomace for the Production of High-Value Bread

**DOI:** 10.3390/foods13030460

**Published:** 2024-02-01

**Authors:** Federica Cardinali, Luca Belleggia, Anna Reale, Martina Cirlini, Floriana Boscaino, Tiziana Di Renzo, Lorenzo Del Vecchio, Natascia Cavalca, Vesna Milanović, Cristiana Garofalo, Cristiana Cesaro, Giorgia Rampanti, Andrea Osimani, Lucia Aquilanti

**Affiliations:** 1 Dipartimento di Scienze Agrarie, Alimentari ed Ambientali, Università Politecnica delle Marche, Via Brecce Bianche, 60131 Ancona, Italy; f.cardinali@univpm.it (F.C.); l.belleggia@pm.univpm.it (L.B.); v.milanovic@univpm.it (V.M.); c.garofalo@univpm.it (C.G.); c.cesaro@pm.univpm.it (C.C.); g.rampanti@pm.univpm.it (G.R.); l.aquilanti@univpm.it (L.A.); 2 Istituto di Scienze dell’Alimentazione, Consiglio Nazionale delle Ricerche, Via Roma 64, 83100 Avellino, Italy; anna.reale@isa.cnr.it (A.R.); floriana.boscaino@isa.cnr.it (F.B.); tiziana.direnzo@isa.cnr.it (T.D.R.); 3 Dipartimento di Scienze degli Alimenti e del Farmaco, Università di Parma, Viale Parco Area delle Scienze 27/A, 43124 Parma, Italy; martina.cirlini@unipr.it (M.C.); lorenzo.delvecchio@unipr.it (L.D.V.); natascia.cavalca@unipr.it (N.C.)

**Keywords:** antioxidant capacity, olive oil by-product, shelf life, bread-making, wholemeal bread, volatilome

## Abstract

In this study, the morpho-textural features, total phenolic content (TPC), and antioxidant capacity (AOC) of bread fortified with olive (*Olea europaea* L.) pomace were evaluated. Fresh olive pomace was subjected to microbiological and chemical (TPC, AOC, and fiber) analyses; then, the same olive pomace was analyzed during 1 to 6 months of storage at 4 °C or −20 °C. All olive pomace samples were used in 10%, 15%, or 20% amounts to produce type 0 soft wheat (*Triticum aestivum*) and whole wheat bread samples. The volatile organic compounds (VOCs) in the bread samples were also analyzed to assess the effect of the addition of the olive pomace on the flavor profile of the baked products. The TPC and AOC evaluation of olive pomace showed no differences among the analyzed samples (fresh, refrigerated, or frozen). Regarding the bread containing olive pomace, the specific volume was not affected by the amount or the storage methods of the added pomace. Bread samples produced with soft wheat flour showed the lowest hardness values relative to those produced with whole wheat flour, irrespective of the amount or storage method of the olive pomace. Regarding color, the crust and crumb of the bread samples containing 20% olive pomace were significantly darker. The bread samples containing 20% olive pomace had the highest TPC. The bread samples with fresh olive pomace were characterized by terpenoids, ketones, and aldehydes, whereas the bread samples containing refrigerated olive pomace were characterized by alcohols (mainly ethanol), acids, esters, and acetate. Finally, the bread samples with frozen olive pomace showed a volatile profile similar to that of bread produced with fresh olive pomace. Olive pomace was shown to be a suitable ingredient for producing bread with high nutritional value.

## 1. Introduction

In the Mediterranean basin, the cultivation of *Olea europaea* L. provides virgin olive oil that, due to its nutritional benefits in the human diet, has been recognized as one of the best vegetable oils since ancient times [1,2]. However, the production of olive oil also engenders large amounts of by-products and wastes, including so-called olive pomace, olive mill waste waters, olive leaves, olive stones, and seeds [1]. Furthermore, the disposal of these by-products and wastes represents a threat to the environment and a further cost for the olive oil food industry [3]. As an example, between 2020 and 2021, more than 8 million tons of olive mill waste waters were produced worldwide [4], with a considerable impact on the environment in terms of phytotoxicity, pollution of natural waters, threat to aquatic life, and offensive odors [5]. Based on the global society’s growing awareness of sustainable food production, the European Union (EU) issued the Circular Economy Action Plan within the European Green Deal. This action, which is a prerequisite for achieving the EU’s 2050 climate neutrality target and halting the loss of biodiversity, is aimed at reducing pressure on natural resources and creating sustainable growth and job opportunities.

In such a context, the olive oil industry has developed new strategies to convert its by-products and wastes into commercially viable products, such as fuels, fertilizers, feed ingredients, compost, cement and bricks, and phytochemicals [4,6,7,8]. As reviewed by Roselló-Soto et al. [9], by-products and wastes from olive oil production include compounds of considerable nutritional value such as fatty acids, pigments (carotenoids and chlorophylls), tocopherols, phytosterols, squalene, volatile and aromatic compounds, and polyphenols. Therefore, academic researchers and the food industry have started to jointly design and promote new high-value food products that incorporate variable amounts of by-products from the olive oil industry to (i) foster circular economy processes, (ii) encourage sustainable consumption, (iii) ensure waste prevention, and (iv) exploit the nutritional value of food by-products.

Among the by-products obtained during olive oil production, olive pomace has already proved to be a very versatile ingredient for enriching conventional food products. Indeed, Ying et al. [10] showed the feasibility of preparing fiber- and polyphenol-enriched extruded food products based on mixed rice–oat flour and maize–oat flour containing olive pomace. Moreover, Balli et al. [11] have successfully used olive pomace to enrich tagliatelle pasta with phenolic compounds and fiber, whereas Simonato et al. [12] observed a positive impact on the glycemic response of pasta containing olive pomace. Interestingly, Ribeiro et al. [13] produced yogurt enriched in fiber and hydroxytyrosol by incorporating olive pomace into milk. Finally, a few authors have exploited olive pomace to produce bakery products including biscuits, taralli, and bread with enhanced nutritional value [14,15,16,17].

Bread represents a staple food in the typical Western diet. In bread-making, refined wheat flour (commonly classified as type 00 or 0) represents the most used raw material; however, bread lacks in essential amino acids and fiber, the latter of which is abundant in bran and germ [18,19]. Conversely, bread produced with whole wheat flour is rich in fiber, thereby conferring health benefits to the consumer including the reduction of glycemic index and blood cholesterol [19]. Notwithstanding, whole wheat flour has a low gluten content, thus producing a bread dough with low technological features (reduced stability, resistance, and extensibility) [19]. To produce bread, the leavening of the dough can be obtained through (i) the direct method, consisting of the addition of baker’s yeast to a mixture of water and flour, or (ii) through indirect methods, which involve the use of either “sourdough” or “sponge” (with the latter also referred to as *biga*) as the leavening agent [20,21]. The *biga* method consists of two steps. The first step produces a light “sponge” by mixing flour, water, and baker’s yeast, followed by ~16 h of fermentation; the second step leads to a “dough” by mixing the remaining ingredients and the *biga*, followed by a final fermentation (leavening) before baking [20].

To date, many studies have focused on improving the nutritional and rheological features of bread produced with new functional ingredients [18]. To the best of the authors’ knowledge, for bread, the added amount of olive pomace usually ranges between 4% [16] and 10% [15], and knowledge of the use of this ingredient in bread-making is still limited. Hence, bread represents an optimal low-cost candidate to valorize olive pomace and obtain a healthy and value-added food.

This study aimed to evaluate the chemical, nutritional, and volatile characteristics of bread fortified with olive pomace. For this purpose, fresh olive pomace obtained from the milling of black olives (*Olea europaea* L. cv. Piantone di Mogliano) was first subjected to microbiological and chemical analyses. The same olive pomace was then stored under different refrigeration conditions and analyzed for the same parameters after 1, 2, 3, 4, 5, and 6 months of storage. Then, fresh olive pomace and that maintained under the abovementioned storage conditions were used at different concentrations to produce experimental bread samples. The doughs were subjected to microbiological analyses, whereas the baked bread samples were subjected to morpho-textural and chemical analyses. As is widely acknowledged, the flavor of bread is influenced by enzymatic reactions during dough fermentation and by thermal reactions occurring during the baking process, such as the non-enzymatic Maillard reactions and the caramelization of sugars. Moreover, the presence and quantity of new ingredients in the bread recipe can influence the aroma of the end product. Hence, to evaluate the influence of the added olive pomace in the flavor profiles of the experimental bread samples, an analysis of volatile organic compounds (VOCs) using solid-phase microextraction (SPME) coupled with gas chromatography–mass spectrometry (GC–MS) was also performed.

## 2. Materials and Methods

### 2.1. Raw Materials

Fresh olive pomace from black olives (*Olea europaea* L. cv. Piantone di Mogliano) was obtained by a three-phase olive oil production process at the Corradini oil mill (Mogliano, Italy). Olive pomace was transported to the laboratory under refrigerated conditions (4 °C) and analyzed immediately for chemical and microbiological characteristics. Then, the fresh olive pomace was vacuum-packed in sterile food-grade plastic bags and stored for 6 months at −20 °C or 4 °C. The stored samples were analyzed after 1, 2, 3, 4, 5, and 6 months to assess their shelf life. No treatment was applied to the raw olive pomace.

**Figure 1:**
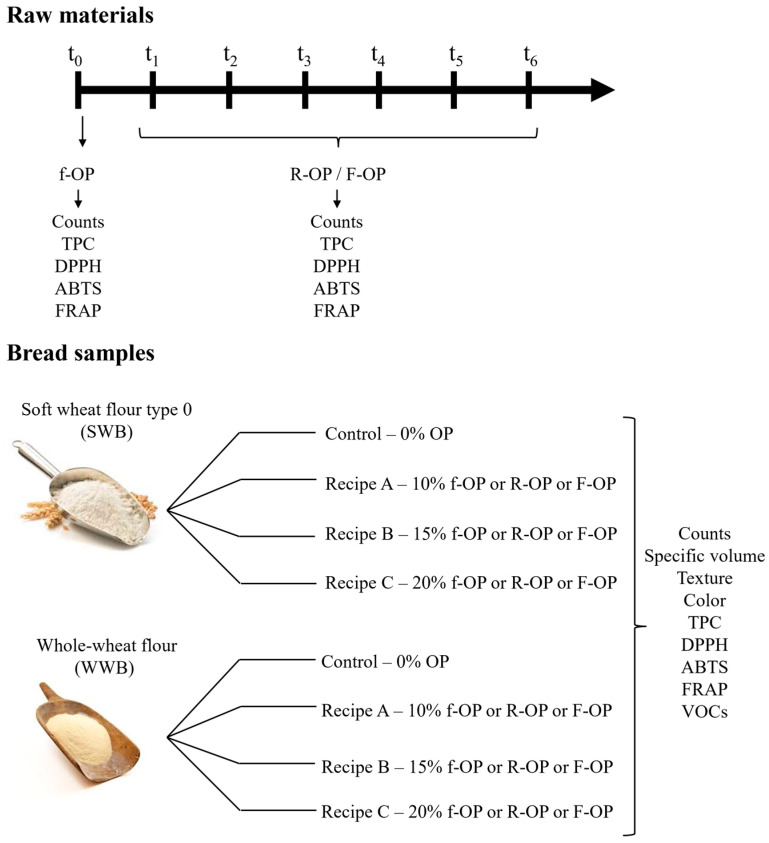
Experimental design. f-OP: fresh olive pomace; R-OP: refrigerated olive pomace; F-OP: frozen olive pomace.

Thereafter, the olive pomace stored for 6 months under the two different conditions was used as an ingredient in the bread-making process, as detailed in Section 2.3. Type 0 soft wheat flour was supplied by Molino Bianchi (Osimo, Italy), whereas whole wheat flour was supplied by Molino Fratini (Pollenza, Italy). The experimental design is shown in Figure 1.

### 2.2. Analyses of Olive Pomace during the 6-Month Storage Period

#### 2.2.1. Microbiological Analyses

For the microbiological analyses, 10 g of fresh or stored olive pomace was added to 90 mL of sterile peptone water (1 g L^−1^) and homogenized in a 400 Circulator Stomacher apparatus (International PBI, Milan, Italy) at 260 rpm for 2 min. Aliquots of 1 mL were serially ten-fold diluted in sterile peptone water for the enumeration of specific microbial groups. In more detail, mesophilic aerobic bacteria were counted in Plate Count Agar (PCA) (VWR International Srl, Milan, Italy) incubated at 30 °C for 48 h; presumptive mesophilic lactobacilli were counted on De Man, Rogosa, and Sharpe (MRS) agar (VWR) supplemented with cycloheximide (250 mg L^−1^) (VWR) incubated for 48–72 h at 37 °C; eumycetes (yeasts and molds) were counted on Rose Bengal Chloramphenicol Agar (RBA) (VWR) incubated for 4 days at 25 °C; and Enterobacteriaceae were counted in Violet Red Bile Glucose Agar (VRBGA) (VWR) incubated for 24 h at 37 °C.

The results were expressed as the mean Log cfu (colony forming units) g^−1^ of two independent analyses ± standard deviation.

#### 2.2.2. Evaluation of Phenolic Fraction

Antioxidant compounds were extracted from the olive pomace samples following the protocol reported by Dall’Asta et al. [22] with some modifications. Briefly, 0.5 g of sample was weighted and added to 5 mL of methanol/water solution (80/20 *v*/*v*) and then extracted on an HS 501 digital shaker (IKA-Werke, Staufen, Germany) at 200 strokes min^−1^ for 30 min at room temperature. Then, the extracts were centrifuged at 5000 rpm for 10 min at room temperature using a Centrifugette 4206 centrifuge (Alc International, Pévy, France), and the supernatants were recovered and kept in the dark at −20 °C until analysis. Each sample was extracted in duplicate.

Total phenolic content (TPC) was evaluated based on the Folin–Ciocalteau method in accordance with the protocol already described by Martin-Diana et al. [23] with a few modifications. In more detail, 250 µL of sample extract was mixed with 1 mL of Folin–Ciocalteau reagent previously diluted in bi-distilled water (1/10 *v*/*v*) and 2 mL of sodium carbonate aqueous solution (10% *w*/*v*) and then kept in the dark for 30 min at room temperature. Then, the absorbance of the solution was measured in triplicate at 760 nm using a V-530 spectrophotometer (JASCO, Easton, MD, USA). To calculate TPC, a calibration curve was constructed by analyzing—under the same conditions—five gallic acid standard solutions (0.01^−1^ mg GAE g^−1^); a blank sample was also analyzed.

#### 2.2.3. Determination of the Antioxidant Capacity (AOC)

##### DPPH Radical-Scavenging Activity Assay

To determine the radical-scavenging capacity of the extracts, the DPPH (2,2-Diphenyl-1-picrylhydrazyl) radical-scavenging assay was applied according to Dall’Asta et al. [22]. In more detail, 100 µL aliquots of each sample extract were mixed with 2.9 mL of a methanolic DPPH solution (0.05 mM) and kept in the dark at room temperature for 30 min. The absorbance of the samples was then recorded in triplicate at 517 nm using a JASCO V-530 spectrophotometer. A blank sample was prepared using 100 µL of extraction solution and then measured after incubation with the DPPH reagent solution. To quantify the antioxidant capacity of the extracts, a calibration curve was prepared using Trolox as the reference standard in the concentration range of 0.025–0.25 mg g^−1^ (five points). The radical-scavenging capacity (I%) was calculated considering the percentage of radical inhibition. The following mathematical formula was applied:

I% = [(Abs_blank_ − Abs_sample_)/Abs_blank_] × 100

where Abs_blank_ is the absorbance of the blank sample and Abs_sample_ is the absorbance of the standard solution or sample. The results were expressed as mg TEAC (Trolox Equivalent Antioxidant Capacity) g^−1^.

##### ABTS Radical-Scavenging Activity Assay

ABTS [2,2-azinobis (3-ethylbenzothiazoline-6-sulfonic acid)] assay was carried out in accordance with the method reported by Jemai et al. [24] with some modifications. For the generation of the ABTS radical cation (ABTS+), an aqueous solution containing ABTS (7 mM) and potassium persulfate (2.45 mM) was prepared and kept in the dark for ~16 h. Then, the solution was fully diluted with ethanol (1.70^−1^ *v*/*v*), and its absorbance at 734 nm was measured using a JASCO V-530 spectrophotometer, checking that the value did not exceed 0.70 ± 0.2. For the photometric assay, 20 µL aliquots of sample extract (either blank or standard solution) were treated with 1.98 mL of the ABTS+-diluted solution. The reaction was performed in the dark at room temperature and, then, the absorbance of all the samples was measured in triplicate at 734 nm. The quantification was performed based on Trolox, as already described in the DPPH determination procedure.

##### Determination of Ferric-Ion-Reducing Power (FRAP)

The antioxidant capacity of the sample extracts was also evaluated using FRAP assay. For this analysis, the FRAP reagent solution was prepared by mixing 2.5 mL of an aqueous solution of ferric chloride hexahydrate (20 mM) with 2.5 mL of an aqueous solution of 2,4,6-tripyridyl-s-triazine (TPTZ) (10 mM) acidified with hydrochloric acid (40 mM) and 25 mL of 300 mM acetate buffer prepared from sodium acetate and acetic acid (pH 3.6) [25]. The solution was heated to 37 °C prior to use. Sample extracts, blank samples, and Trolox standard solutions (150 µL) were submitted to the reaction with the FRAP solution (2.85 mL) in the dark at room temperature for 30 min; then, the absorbance was measured in triplicate at 593 nm using a JASCO V-530 spectrophotometer. The ferric-ion-reducing activity in the samples was estimated based on Trolox, by constructing a calibration curve in the same concentration range used in the previously described tests.

### 2.3. Bread-Making Trials

Bread-making trials were carried out in duplicate using fresh, frozen-stored (−20 °C), or refrigerated-stored (4 °C) olive pomace. For each trial, bread loaves were produced according to the recipes reported in Table 1.

First, a *biga* was prepared by mixing 67.50% (*w*/*w*) type 0 soft wheat flour, 31.5% (*v*/*w*) water, and 1% (*w*/*w*) fresh baker’s yeast, which was left to ferment at 18 °C for 18 h.

For each trial, all the ingredients and the *biga* were mixed for 15 min in a Model K12-2V spiral mixer (Kosmitech Srl, Morrovalle, Italy) and the doughs were divided into portions of 300 g. After 1.5 h of proofing at 30 °C and 70% relative humidity (R.H.), the doughs were baked in a Model FOSTR6 electric oven (Fimar S.p.a., Villa Verucchio, Italy) at 200 °C for 1 h. Bread loaves without olive pomace were used as controls.

#### 2.3.1. Analyses of Dough and Bread Samples

##### Microbiological Analyses

For the microbiological analyses, 10 g dough samples collected before and after leavening were added to 90 mL of sterile peptone water and homogenized in a Stomacher apparatus (International PBI) for 2 min at 260 rpm. Aliquots of 1 mL were serially ten-fold diluted in sterile peptone water for the enumeration of presumptive lactobacilli on MRS agar (VWR) supplemented with cycloheximide (250 mg L^−1^) (VWR) and incubated at 30 °C for 48–72 h and eumycetes on RBA (VWR) incubated at 25 °C for 4 days. For the viable counts of the bread samples, 10 g aliquots were used for the enumeration of bacterial spores in PCA medium (VWR). The results were expressed as the mean of the Log cfu g^−1^ of two independent measurements ± standard deviation.

##### Determination of Bread Specific Volume, Hardness, and Color

Bread volume was determined using the AACC 10-05.01 rapeseed displacement method; the specific volume, expressed as cm^3^ g^−1^, was calculated as the ratio between the loaf bread volume and loaf weight. All determinations were performed in triplicate and the results were expressed as the mean value ± standard deviation.

The hardness of the bread was measured with a Texture Analyzer (model CT3-4500, Brookfield Engineering Laboratories Inc., Middleboro, MA, USA) using a 36 mm diameter cylindrical probe (mod. TA-AACC36). A 4500 g load cell was used. The probe compressed the crumb to a 40% compression limit (10 mm compression depth) at a speed of 100 mm min^−1^. The measurements were performed at room temperature in the middle of bread slices (20 mm thickness). For each sample, three independent measurements were carried out.

The color measurements were performed using a Chroma Meter CR-200 (Minolta, Osaka, Japan) with a D65 illuminant. Color parameters were determined only for the bread samples produced with fresh olive pomace, which were used as a reference for future product development. In more detail, the color of the crust and crumb was determined according to the CIE L*a*b* system (L*, brightness; a*, redness/greenness; b*, blueness/yellowness) [26]. Bread slices were analyzed by cutting them longitudinally (10 mm thickness) and reproducing their cross sections using a scanner (ENVY 6200 Series, HP, Palo Alto, CA, USA) [27].

##### Evaluation of Bread Phenolic Fraction

Antioxidant compounds were extracted from the bread samples following the protocol reported by Dall’Asta et al. [22] with the same modifications already described in Section 2.2.2.

The TPC determination of the bread samples was carried out as already described in Section 2.2.2.

The determinations of the AOC, DPPH radical-scavenging activity assay, the ABTS radical-scavenging activity assay, and the FRAP of bread samples were carried out as already described in Section 2.2.3.

##### Evaluation of Bread Dietary Fiber

Dietary fiber content was determined using the AOAC 991.43 enzymatic–gravimetric method.

##### SPME–GC/MS Analysis of Bread Volatile Components

The volatile fractions of the breads were analyzed using headspace sampling based on the solid-phase microextraction technique (SPME) according to Cardinali et al. [28]. In detail, for each SPME analysis, 2 g of sample was placed into a 20 mL headspace vial, and 5 μL of 4-methyl-2 pentanol (internal standard, 100 mg L^−1^ standard solution) was added. Each vial was placed in a thermostatic block (40 °C) on a stirrer, and the fiber was inserted and maintained in the sample headspace for 30 min. Then, the fiber was removed and immediately inserted into the GC/MS injector for the desorption of compounds. The extraction was automatically performed by the multipurpose sampler of the GC/MS system.

For the analyses, a silica fiber coated with 75 μm of Carboxen/Polydimethylsiloxane (CAR/PDMS) was used (Supelco, Bellefonte, PA, USA). The SPME–GC/MS analysis was performed using an Agilent GC 7890A/MSD 5975 system with an automatic sampler Gerstel MPS2 (Agilent Technologies, Santa Clara, CA, USA). The experimental setup and operating conditions were as follows: an HP-Innowax capillary column was used (Agilent Technologies, 30 m × 0.25 mm ID, film thickness 0.25 μm), the gas carrier was helium (flow rate = 1.5 mL min^−1^), and SPME injections were splitless (straight glass line, 0.75 mm I.D.) at 240 °C for 20 min, during which time thermal desorption of the analytes from the fiber occurred. The oven parameters were as follows: the initial temperature was 40 °C, held for 3 min, followed by an increase to 240 °C at a rate of 5 °C min^−1^, and then held for 10 min. The injector temperature was 240 °C. The mass spectrometer was operated in scan mode over a mass range of 33 to 300 amu (2 s scan^−1^) at an ionization potential of 70 eV. VOC identification was achieved by comparing the mass spectra with the Nist library (NIST 20) and by matching the retention indices (RI) calculated according to the equation of Van Den Dool and Kratz [29] and based on a series of alkanes. Data were expressed as the relative peak area with respect to the internal standard. Blank experiments were conducted in two different modalities—blank fiber and blank empty vial. These controls were carried out after every 10 analyses. The analyses were performed in duplicate.

### 2.4. Statistical Analysis

A one-way analysis of variance (ANOVA) was applied to compare the data (IBM SPSS Statistics 26.0 software, Chicago, IL, USA). In more detail, for the olive pomace samples, the one-way ANOVA test was used to compare the results obtained among the refrigerated sample or frozen sample groups, whereas, for the bread, all the samples were included in the same dataset. Moreover, a *t*-test for independent samples was used to compare the data obtained from the refrigerated and frozen olive pomace samples, considering a *p*-value of 0.05 as statistically significant. Finally, the correlations among the results obtained from the different assays were also tested using a two-tailed Pearson correlation analysis.

To evaluate how the different breads were distributed according to the detected chemical groups of volatile compounds, a Principal Component Analysis (PCA) was performed using Tanagra 1.4 software.

## 3. Results

### 3.1. Microbiological Analyses of Olive Pomace

The results of the viable counts of the olive pomace samples are reported in Table 2.

In more detail, total mesophilic aerobes were counted at 5.00 ± 0.12 Log cfu g^−1^ in the fresh olive pomace. During storage, the counts of the refrigerated olive pomace varied between 5.02 ± 0.04 (t_1_) and 1.69 ± 0.12 Log cfu g^−1^ (t_5_), whereas the counts of the frozen olive pomace varied between 3.59 ± 0.03 (t_1_) and 2.89 ± 0.06 Log cfu g^−1^ (t_6_), with a progressive and significant decrease from t_1_ to t_6_.

Regarding lactic acid bacteria, for the fresh olive pomace, counts were 2.96 ± 0.01 Log cfu g^−1^. During storage, viable counts of the refrigerated olive pomace varied between 1.87 ± 0.04 (t_1_) and 1.86 ± 0.03 Log cfu g^−1^ (t_4_), whereas for frozen olive pomace, the viable counts varied between 2.13 ± 0.07 (t_1_) and 2.08 ± 0.05 Log cfu g^−1^ (t_6_), with no significant differences among the samples.

In the fresh olive pomace, eumycetes were counted at 4.81 ± 0.15 Log cfu g^−1^. In the refrigerated olive pomace, the counts of eumycetes ranged between 5.69 ± 0.02 (t_1_) and 6.54 ± 0.00 Log cfu g^−1^ (t_6_), whereas the counts in frozen olive pomace ranged between 3.33 ± 0.02 (t_1_) and 3.08 ± 0.01 Log cfu g^−1^ (t_6_), with no significant differences among the samples.

Finally, all samples of olive pomace showed viable counts of Enterobacteriaceae of <1 Log cfu g^−1^.

### 3.2. Total Phenolic Content (TPC) and Antioxidant Capacity of Olive Pomace

The results of the total phenolic content and antioxidant capacity analyses of the olive pomace samples are reported in Table 3.

The TPC of the fresh olive pomace was 7.18 ± 0.12 mg GAE g^−1^; no significant differences were observed among the values of the fresh olive pomace and refrigerated or frozen olive pomace during storage. In more detail, the TPC of the refrigerated olive pomace ranged between 7.35 ± 0.16 (t_1_) and 7.39 ± 0.28 mg GAE g^−1^ (t_6_), whereas the TPC values of the frozen olive pomace ranged between 8.40 ± 0.59 (t_1_) and 9.05 ± 0.29 mg GAE g^−1^ (t_6_).

Regarding antioxidant capacity, the fresh olive pomace samples yielded values of 16.21 ± 0.41 mg TEAC g^−1^ when the DPPH radical was used (corresponding to 64.68 ± 1.62 mmol Trolox kg^−1^), 15.95 ± 0.19 mg TEAC g^−1^ when applying the FRAP method, and 15.36 ± 0.51 mg TEAC g^−1^ using the ABTS assay. The antioxidant capacities measured using the three different applied assays showed a significant, positive Pearson correlation with TPC (DPPH *p* = 0.924; ABTS *p* = 0.561; FRAP *p* = 0.933). The results obtained for the refrigerated and frozen samples were compared to the values obtained for the fresh olive pomace, and, at the same time, a comparison among the two storage temperatures (+4 °C and −20 °C) was also evaluated. Based on the one-way ANOVA, the refrigerated and frozen samples maintained the initial characteristics in terms of TPC and antioxidant capacity over time, especially when comparing t_0_ with the prolonged storage time. In addition, a *t*-test for independent samples was used to determine differences among the two storage methods (refrigerated or frozen). By comparing the results obtained from the refrigerated and stored samples for the four applied tests, statistically higher TPC and AOC values were observed for the frozen olive pomace, especially at the end of the storage period.

### 3.3. Microbiological Analyses of Dough and Bread

The results of the viable counts of lactobacilli and eumycetes in the dough samples, before and after leavening, produced with the addition of different amounts (10%, 15%, or 20%) of fresh, refrigerated, or frozen olive pomace are reported in Table 4.

For the dough made with fresh olive pomace produced with type 0 soft wheat flour, the viable counts of lactobacilli before leavening ranged between 1.83 ± 0.18 and 2.69 ± 0.04 Log cfu g^−1^ with a 0% or 20% ratio, respectively, with statistically significant differences among the samples. After leavening, the lactobacilli count ranged from 2.02 ± 0.09 to 3.04 ± 0.33 Log cfu g^−1^ in the doughs made with fresh olive pomace with a 0% or 20% ratio, respectively, with no statistically significant differences among the samples. Regarding the dough produced with the fresh olive pomace and whole wheat flour, the viable counts of lactobacilli before leavening ranged between 1.72 ± 0.17 and 3.04 ± 0.10 Log cfu g^−1^ with a 0% or 20% pomace ratio, respectively, with statistically significant differences among the samples. After leavening, the lactobacilli counts ranged from 1.39 ± 0.12 to 3.02 ± 0.13 Log cfu g^−1^ with a 0% or 20% pomace ratio, respectively, with statistically significant differences among the samples. No differences were observed among the lactobacilli counts of the analyzed samples before or after leavening, irrespective of the added amount of fresh olive pomace or flour used.

As for the dough with refrigerated olive pomace produced using type 0 soft wheat flour, the viable counts of lactobacilli before leavening ranged between 1.48 ± 0.40 and 3.48 ± 0.13 Log cfu g^−1^ with a 0% or 20% pomace ratio, respectively, with statistically significant differences among the samples. After leavening, the lactobacilli count ranged from 1.50 ± 0.24 to 3.66 ± 0.20 Log cfu g^−1^ in the dough made with fresh olive pomace at a 0% or 20% ratio, respectively, with statistically significant differences among the samples. Regarding the dough produced with refrigerated olive pomace and whole wheat flour, the viable counts of lactobacilli before leavening ranged between 1.86 ± 0.45 and 3.69 ± 0.66 Log cfu g^−1^ with a 0% or 20% pomace ratio, respectively, with statistically significant differences among the samples. After leavening, the lactobacilli counts ranged from 2.32 ± 0.22 to 3.34 ± 0.77 Log cfu g^−1^ with a 0% or 20% pomace ratio, respectively, with no statistically significant differences among the samples. No differences were observed among the lactobacilli counts of the analyzed samples before or after leavening, irrespective of the added amount of fresh olive pomace or flour used.

As for dough with the frozen olive pomace produced with type 0 soft wheat flour, the viable counts of lactobacilli before leavening ranged between 1.48 ± 0.40 and 1.15 ± 0.30 Log cfu g^−1^ with a 0% or 20% pomace ratio, respectively, with no statistically significant differences among these samples. After leavening, the lactobacilli counts ranged from 1.50 ± 0.24 to 1.80 ± 0.55 Log cfu g^−1^ in the dough made with fresh olive pomace with a 0% or 20% pomace ratio, respectively, with no statistically significant differences among these samples. Regarding the dough produced with refrigerated olive pomace and whole wheat flour, the viable counts of lactobacilli before leavening ranged between 1.86 ± 0.45 and 2.60 ± 0.96 Log cfu g^−1^ with a 0% or 20% pomace ratio, respectively, with no statistically significant differences among the samples. After leavening, the lactobacilli count ranged from 2.32 ± 0.22 to 1.83 ± 1.59 Log cfu g^−1^ with a 0% or 20% pomace ratio, respectively, with no statistically significant differences among the samples. No differences were observed among the lactobacilli counts of the analyzed samples before or after leavening, irrespective of the added amount of olive pomace or flour used.

Eumycetes were counted to assess the viability of the baker’s yeast used to allow leavening, and data are reported here in brief. For this microbial group, counts between 6.20 ± 0.01 and 7.41 ± 0.14 Log cfu g^−1^ were obtained, with a few samples showing statistically significant differences.

Finally, no bacterial spores were detected in any of the experimental bread samples that were analyzed soon after baking.

### 3.4. Specific Volume, Hardness, and Color of the Experimental Breads

The results of the specific volume assessments of experimental bread loaves produced with different amounts of olive pomace are reported in Table 5.

In more detail, for the bread produced with type 0 soft wheat flour, no statistically significant differences were observed among the samples, irrespective of the olive pomace inclusion amounts or storage methods. Similarly, for the bread produced with whole wheat flour, no statistically significant differences were observed among the samples, irrespective of the olive pomace inclusion amounts or storage methods.

The bread samples produced with soft wheat flour generally showed no statistically significant differences in specific volume compared to those produced with whole wheat flour without, except those without olive pomace. In these latter samples, the bread produced with soft wheat flour showed the highest average specific volume values.

The results of the hardness measurements of the experimental bread loaves are reported in Table 5.

In more detail, the samples produced with soft wheat flour and 0% fresh olive pomace showed the highest values; the same trend was observed for the bread samples produced with wholemeal wheat flour and 0% olive pomace. As for the samples produced with soft wheat flour and refrigerated olive pomace, the highest hardness value was observed in the bread with 0% olive pomace, whereas, for the bread made with whole wheat flour, significant differences were observed among the samples, with the sample containing 10% olive pomace showing the lowest average value.

Regarding the bread produced with soft wheat flour and frozen olive pomace, the highest hardness values were observed in samples containing 15% and 20% olive pomace; the same trend was observed for the bread samples produced with whole wheat flour.

The bread samples produced with soft wheat flour showed the lowest hardness values compared to those produced with whole wheat flour, irrespective of the amount or storage method of the olive pomace.

**Figure 2:**
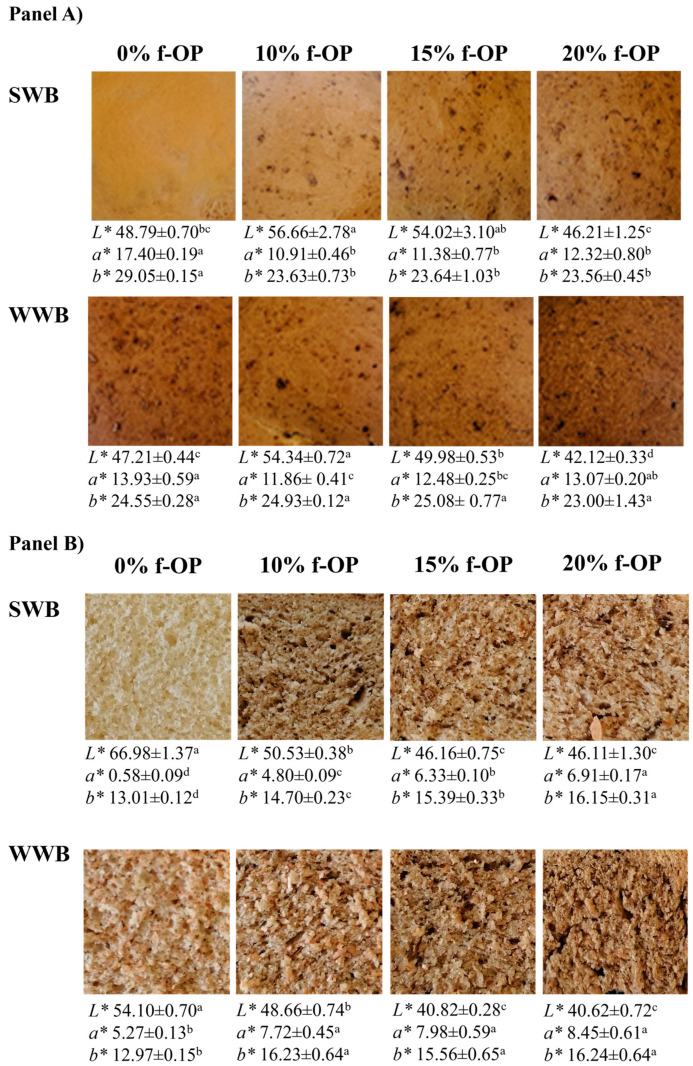
Images of the crust (panel **A**) and crumb (panel **B**) of bread samples containing fresh olive pomace (f-OP) produced with soft wheat flour type 0 (SWB) or whole wheat flour (WWB). Means ± standard deviations of triplicate independent measurements are shown. Within each panel and each type of bread (SWB or WWB), for the same color parameter, means followed by different letters are significantly different (*p* < 0.05). L* values describe the lightness; a* values describe the redness/greenness; b* values describe the blueness/yellowness.

Finally, the color parameters evaluated for bread loaves produced with fresh olive pomace are reported in Figure 2.

For the crust of the bread loaves produced with soft wheat flour, the average value of the L* parameter was the lowest in the samples containing 20% olive pomace. The average values of the a* and b* parameters were the highest in the samples produced with no olive pomace. The same differences were observed for the crust of the bread loaves made with whole wheat flour.

As for the crumb of the bread loaves made with soft wheat flour, the average value of the L* parameter was the highest in the samples made with no olive pomace. The average values of the a* and b* parameters were the highest in the samples produced with 20% olive pomace. The same differences were observed for the crumb of the bread loaves made with whole wheat flour.

### 3.5. Total Phenolic Content, Antioxidant Capacity, and Dietary Fiber of the Breads

The total phenolic content, antioxidant activity, and dietary fiber values of the bread samples studied in this research are reported in Table 6.

As expected, the bread produced with type 0 soft wheat flour resulted in samples with the lowest TPC; the whole wheat bread showed slightly higher TPC values than those of the bread produced with soft wheat flour, although this difference was not significant. The addition of higher percentages of olive pomace in the bread dough led to an augmentation of the TPC for both the bread types, and the highest values were measured in the bread samples containing 20% olive pomace.

The antioxidant activity of the tested samples reflected the concentrations of polyphenols found in the same products. This was proved by calculating the Pearson correlation coefficient among the four datasets (TPC, DPPH, ABTS, and FRAP values) and obtaining a high value, ranging between 0.845 and 0.969. A positive relationship between the phenolic amount and the antioxidant properties of the samples was observed, with a strong relationship among the different assays applied in the AOC determination. The bread samples without olive pomace had significantly lower AOC, whereas those containing 20% olive pomace showed the highest AOC.

As for dietary fiber, the bread samples produced with type 0 soft wheat flour and without olive pomace showed statistically significant lower values than those containing different percentages of olive pomace. In more detail, the bread made with soft wheat flour and 20% olive pomace showed the highest values, irrespective of the pomace storage conditions. In the bread samples produced with whole wheat flour, no significant differences were observed, irrespective of the pomace storage conditions or the percentage of olive pomace added. Finally, for the samples without olive pomace (control), those produced with soft wheat flour showed the lowest dietary fiber content compared to those containing whole wheat flour. No significant differences emerged among the samples containing 10% to 20% olive pomace, irrespective of the flour type used.

### 3.6. SPME–GC/MS Analysis of the Volatile Components of the Breads

The SPME–GC/MS analysis allowed the volatile profiles of the bread samples to be obtained for those made with type 0 soft wheat (Table 7) and whole wheat (Table 8) flours and with the addition of different amounts of olive pomace stored under different conditions.

The detected compounds belonged to nine classes— ketones (7), aldehydes (6), alcohols (5), acids (5), furans and pyrans (5), pyrazines (5), esters and acetates (4), terpenoids (3), and phenols (2).

Among the carboxylic acids, acetic acid was the most represented type in all the samples. Beta pinene, limonene, and copaene were the detected terpenoids.

Among the phenols, vinylguaiacol and guaiacol were found in trace amounts.

Among the furans, 2-pentylfuran, 2-furfural, and 2-furanmethanol were detected in almost all of the samples and at the highest amounts.

Regarding the alcohols, ethanol was the most detected volatile compound in all the samples followed by isoamylalcohol, isobutanol, and 1-hexanol.

Among the acetates and esters, ethyl acetate was found in the highest amounts in all the samples.

Methylpyrazine, 2,3-dimethylpyrazine, and 2-ethyl-5-methylpyrazine were the most detected compounds among the pyrazines.

2-methylbutanal and 3-methylbutanal were the most abundant aldehydes followed by hexanal and benzaldehydes.

Acetoin was the most detected ketone in all the samples.

**Figure 3:**
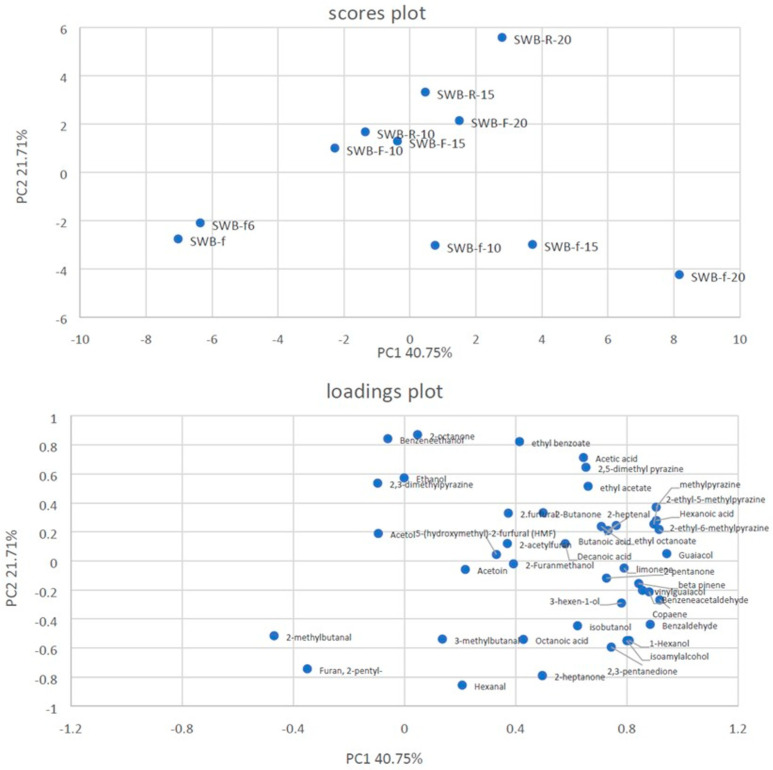
Principal Component Analysis (PCA) of volatile compounds in type 0 soft wheat flour bread (SWB) samples with different concentrations of olive pomace (0%, 10%, 15%, or 20%) stored under different conditions (f, fresh; F, frozen; R, refrigerated).

**Figure 4:**
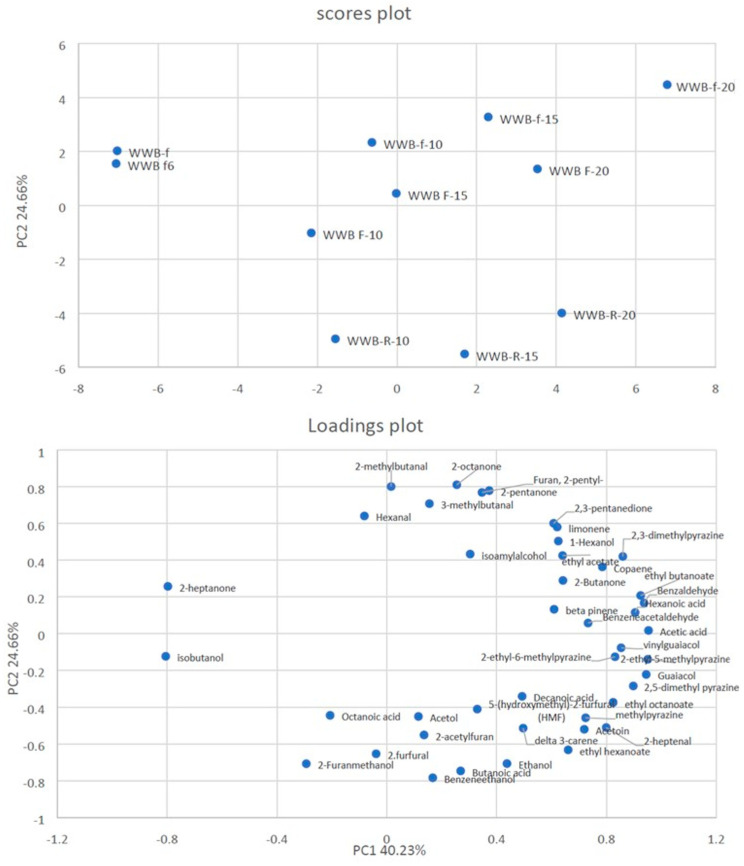
Principal Component Analysis (PCA) of volatile compounds in whole wheat flour bread (WWB) samples with different concentrations of olive pomace (0%, 10%, 15%, or 20%) stored under different conditions (f, fresh; F, frozen; R, refrigerated).

To better understand the differences among the bread samples, PCA was applied to the volatile compounds detected in the type 0 soft wheat bread (Figure 3 and Table 9) and the whole wheat bread (Figure 4 and Table 10) samples.

Regarding the bread samples produced with type 0 soft wheat flour, the two PCs explained 62.46% of the total variance in the data. The samples were located in three different zones of the plot plane. Regarding the score plot, a clear separation between the SWB-f and SWB-f_6_ bread samples produced without olive pomace (negatively associated with PC1) and all the other bread samples produced with different concentrations of olive pomace (positively associated with PC1) was evident.

On the other hand, some differences were also found between samples SWBf-10, 15, and 20 produced with fresh olive pomace (negatively associated with PC2) and with refrigerated and frozen olive pomace (positively associated with PC2).

In particular, the bread samples produced with fresh olive pomace—irrespective of the pomace concentration used—were characterized by terpenoids, phenols, ketones, and aldehydes, whereas the bread samples produced with the refrigerated and frozen olive pomace were characterized by the occurrence of alcohols (mainly ethanol), acids (mainly acetic acid), esters, and acetate.

Figure 4 shows the PCA plot obtained by analyzing the volatile compounds from the bread samples produced with whole wheat flour. The two PCs explained 64.89% of the total variance in the data. The samples were located in three different zones of the plot plane. Also in these samples, regarding the score plot, those produced without olive pomace (negatively associated with PC1) differed from all those produced with different concentrations of olive pomace (positively associated with PC1).

On the other hand, marked differences were also found between the samples produced with fresh olive pomace (positively associated with PC2) and those produced with refrigerated olive pomace (negatively associated with PC2). The bread samples produced with frozen olive pomace plotted close to those produced using fresh olive pomace.

In more detail, the bread samples produced with fresh olive pomace—irrespective of the pomace concentration used—were characterized by terpenoids, ketones, and aldehydes, whereas the bread samples produced with refrigerated olive pomace were characterized by alcohols (mainly ethanol), acids, esters, and acetate. The bread samples produced using frozen olive pomace had a volatile profile that was similar to that of the bread produced with fresh olive pomace.

## 4. Discussion

The use of olive pomace in bread formulation could represent an innovative and low-cost strategy to produce healthy and value-added food products.

However, two important considerations regarding this ingredient must be made. First, the amount of olive pomace can strongly affect the techno-functional and volatile features of bread; second, the seasonality of olive pomace can reduce the exploitability of this ingredient by the food industry. Hence, the added amount of olive pomace should be carefully investigated in order to allow the food industry to produce marketable bread. Moreover, effective preservation methods for olive pomace should be investigated to provide a continuous supply to the food industry.

Based on the abovementioned considerations, in this study, olive pomace maintained under different storage conditions was tested for bread-making in proportions up to 20%, providing the food industry with sound results to inform product development and quality assessment.

As for the microbial loads detected in the fresh olive pomace, a progressive and remarkable reduction in the counts of mesophilic aerobes and lactic acid bacteria was observed in the samples stored at +4 °C, whereas a lower reduction was observed in those stored under frozen conditions. Since the analyzed olive pomace samples were obtained after no microbial stabilization (e.g., heat treatment), the presence of mesophilic aerobes is the result of the environmental (e.g., dust or soil) contamination of the milled olives. Of note, the antioxidant compounds detected in olive pomace can strongly modulate the viability of microorganisms, thus producing a negative selective pressure towards certain taxa (e.g., Enterobacteriaceae) or a positive enhancement of others (e.g., lactic acid bacteria and yeasts) [30,31]. Moreover, it is supposed that storage under frozen conditions also guarantees a higher microbial survival time, thus explaining the higher counts after 6 months at −20 °C.

The results obtained from the analysis of the total polyphenolic content of olive pomace at t_0_ were in accordance with the concentrations of polyphenols found in fresh olives by Piscopo et al. [32], who indicated a TPC of 13.64 ± 0.64 mg GAE g^−1^ for fresh fruits. In addition, the antioxidant activity of the olive pomace samples was determined by means of three different assays (DPPH, ABT, and FRAP). The values obtained for the initial pomace are higher than those reported for green and black olives by Pellegrini et al. [33]. These authors [33] state an antioxidant capacity of 10.43 ± 7–14.73 ± 3 mmol Trolox kg^−1^ as determined using the DPPH test, and 24.59 ± 6–39.99 ± 4 mmol Fe^2+^ kg^−1^ as determined using the FRAP assay for green and black olives, respectively, whereas the samples analyzed herein yielded values of 16.21 ± 0.41 mg TE g^−1^ when DPPH reagents were used (corresponding to 64.68 ± 1.62 mmol Trolox kg^−1^) and 15.95 ± 0.19 mg TE g^−1^ when applying the FRAP method (corresponding to 63.72 ± 0.75 mmol Trolox kg^−1^). The significant and positive Pearson correlation coefficient between the TPC concentrations and AOC values demonstrates that, as expected, the radical scavenger activity (as determined using the DPPH and ABTS tests) and the ferric-reducing ability (as evaluated using the FRAP assay) of olive pomace can be ascribed to polyphenols.

The results obtained for the refrigerated and frozen pomace samples were compared to those obtained for fresh olive pomace and, at the same time, an additional comparison between the samples stored at 4 °C and −20 °C was performed. As a general trend, both the refrigerated and frozen samples maintained their initial characteristics in terms of TPC and antioxidant capacity over time, especially when comparing the t_0_ results with those after the longest storage time. In addition, a *t*-test for independent samples was used to evaluate the differences among the two storage methods. Comparing the results obtained for the refrigerated and frozen pomace samples for the four applied tests, it was possible to observe that the frozen material had a statistically higher TPC and AOC than the refrigerated material. Hence, based on these results, storage at −20 °C seems to represent the best strategy for preserving the initial polyphenolic and antioxidant contents of olive pomace.

The addition of olive pomace to bread led to an increase in its total polyphenolic content, irrespective of the flour used, and this behavior was particularly evident in the bread samples containing 20% olive pomace. These results are in accordance with data obtained by Marinopoulou et al. [34], who studied the phenolic concentrations in bread fortified with green and black olive pulp. Indeed, Marinopoulou et al. [34] observed an increase in phenolic amounts in products to which the highest percentage of olive pulp was added. The addition of a naturally polyphenol-rich matrix in wheat bread recipes may indeed lead to a greater quantity of these compounds in the end product. This is reported also for bread fortified with grape-seed extract [35] as well as for crackers prepared with the addition of microalgae [36]. Concerning the antioxidant properties of the bread samples, similar behavior was observed; high antioxidant capacity values were obtained for the bread samples containing olive pomace. These results are in accordance with data obtained in a previous study in which the influence of adding chestnut flour to a bread recipe was tested, leading to an increase in the antioxidant capacity of the bread [22]. Furthermore, Marinopoulou et al. [34] identified that bread containing green and black olive pulp has higher antioxidant activity than bread without olive supplementation, as is the case for the samples studied herein. Thus, the results of the present study support the exploitation of an olive-based substrate to improve the nutritional quality of bread, both in soft and whole wheat types.

Bread samples incorporating olive pomace exhibited a notable fiber content. Of note, the fiber content of the experimental bread samples containing 20% olive pomace and soft wheat flour was above the level recommended by the European Food Safety Authority (EFSA) for foods classified as “high in fiber”, which corresponds to 6 g of dietary fiber per 100 g of product [37]. This level of fiber was also generally observed in the bread samples made with whole wheat flour, irrespective of the amount of olive pomace. This result is likely due to the presence of wheat bran in these latter bread samples. It is important to note that the EFSA considers a daily intake of 25 g of dietary fiber to be sufficient for normal laxation in adults as well as for reducing the risk of coronary heart disease and type 2 diabetes and for aiding weight maintenance [37]. Based on these considerations, the consumption of bread enriched with olive pomace could effectively contribute to reaching such a daily threshold.

Based on the results, the specific volume of the bread loaves was not influenced by the addition of olive pomace, irrespective of the wheat flour (type 0 soft or whole) used for bread-making or the storage conditions of the olive pomace. These results are in accordance with those obtained by Cedola et al. [15], who did not observe appreciable differences in the rheological features of bread loaves produced with the addition of 10% olive pomace compared with control loaves.

As expected, the bread loaves studied in the present research significantly differed in their crust and crumb color attributes according to the type of wheat flour (type 0 soft or whole) and the amount of olive pomace used in the bread-making. Notwithstanding, the effect of olive pomace addition was less evident in the bread samples containing whole wheat flour, likely due to the dark appearance of loaves with the presence of wheat bran. The results obtained in the present study are in accordance with those obtained by Cedola et al. [15], who reported that bread samples containing olive pomace were darker in color compared to control loaves without this by-product.

Regarding the different color descriptors, lightness can vary from 0 (black) to 100 (white); hence, the progressive reduction of lightness detected in the samples containing olive pomace reflects the increasing quantities of pigments derived from the olives. As for the a* parameter, this axis represents the green–red opponent colors, with values < 0 more green and values > 0 more red. In the present study, all the samples had values in the red hue, except for the control bread produced with 100% type 0 soft wheat flour, which yielded an a* value of 0, thus confirming the strong effect of the addition of olive pomace on bread color. Concerning the b* parameter, this axis represents the blue–yellow opponents, with values < 0 more blue and those > 0 more yellow. In the present study, the b* values of all samples were in the yellow hue range, again reflecting a strong effect of the addition of olive pomace, with the bread produced with 100% type 0 wheat flour resulting in the lowest average yellow levels.

As for the bread-making trials, the microbial counts performed on the doughs show that the effect of olive pomace addition varied according to the added amounts; however, no effect on the leavening (fermentation) ability of the yeast (*S. cerevisiae*) was observed between the samples, as was evidenced by Foti et al. [30]. Of note, the high eumycetes counts detected in all of the analyzed bread doughs attest to the viability of the baker’s yeast (*S. cerevisiae*) used as a leavening agent. Although statistically significant differences were observed among a few dough samples, the numerical differences have no significance from a biological point of view, as the counts of eumycetes at the end of leavening assured proper dough development in all trials. Hence, it is likely that the quantity of phytochemicals carried by the olive pomace did not inhibit yeast performance.

As for spore-forming bacteria, this group of microorganisms includes those that can be the causative agents of bread spoilage (e.g., *Bacillus subtilis*) as well as potential human pathogens (e.g., *Bacillus cereus*, sulfite-reducing clostridia, etc.) [38]. Of note, the absence of these bacteria in the experimental bread samples attests that the baking process was properly performed.

The results obtained in this study show that the addition of olive pomace significantly influences the volatile profile of bread, thus representing an advancement in knowledge. In fact, the bread samples obtained with the addition of olive pomace were characterized by higher amounts of terpenoids, mainly beta pinene and copaene, and phenols such as vinylguaiacol and guaiacol than those without pomace. These compounds are likely derived from the raw olive pomace.

The bread samples produced with olive pomace were also characterized by the highest amounts of furans as 2-furfural and 2-furanmethanol, which confer the aromas of toasted caramel and nuts to bread [39], and the highest amounts of pyrazine. As also highlighted by de Gennaro et al. [40], pyrazines are usually formed by the interaction between the products of Maillard reactions and Strecker degradation and, together with furans, significantly contribute to the flavor of baked products.

The results concerning hexanal are very interesting. Indeed, this compound is a representative marker of oxidative rancidity and could be used as an alternative to traditional oxidation indicators (e.g., acidity or peroxide values) [17]. As ascertained by different authors, the lipid fraction of semolina—the main ingredient of bakery products—is very susceptible to lipoxygenase activity, leading to hydroperoxide production [41]. Hydroperoxides are highly unstable and are converted into volatile compounds, such as hexanal, which are responsible for rancid off-flavors [42]. In fact, the presence of hexanal has already been reported in several cereal-based foods, including pasta, bread, and biscuits. In the present study, hexanal was found in high amounts in the samples without olive pomace, indicating that olive pomace has a protective effect against rancidity; in all the bread samples produced without the addition of olive pomace, the amount of hexanal was higher compared to those containing olive pomace.

The bread samples produced with olive pomace were also characterized by the presence of high levels of acids (acetic, butanoic, and hexanoic acid), likely derived from the fresh olive pomace.

Furthermore, the final volatile profile of the bread samples was also influenced by its storage conditions. The results herein highlight that bread produced with frozen olive pomace shows a volatile profile that is more similar to bread made with fresh olive pomace compared to bread made with refrigerated olive pomace. Hence, it is likely that refrigeration temperatures are not able to slow down the biochemical or enzymatic activities that ultimately affect the finished product.

## 5. Conclusions

In the present study, the suitability of olive pomace for the production of high-value bread was ascertained. It is noteworthy that the storage conditions of the tested olive pomace (fresh, refrigerated, or frozen) did not affect the TPC and antioxidant capacity of the resulting bread, which represents an advance in knowledge regarding the potential use of this olive by-product. The addition of olive pomace, in proportions up to 20%, allowed for the production of bread with increased TPC and with no remarkable influence on the specific volume of the final product. Of note, the olive pomace added to the bread doughs strongly characterized the volatilome component of the loaves, which contained high amounts of terpenoids. The overall results contribute to opening new income opportunities for farmers and the food industry based on more sustainable agriculture in the European Union. Such opportunities aim to reduce food waste and create added value, as expressly stated in the European Green Deal for improving the well-being and health of citizens and future generations. The present research could serve as best practice in the preparation of high-value bread containing olive pomace. Further research is needed to assess the bio-accessibility and functionality of bread bioactive compounds. Finally, consumer tests should be performed in order to evaluate consumer acceptance of this novel high-value bread.

## Figures and Tables

**Table 1:** Recipes for the bread-making process for bread containing olive pomace.

Raw Material	Control		Recipe A		Recipe B		Recipe C	
	SWB	WWB	SWB	WWB	SWB	WWB	SWB	WWB
Olive pomace	0%		10.0%		15.0%		20.0%	
Whole wheat flour		38.2%		31.7%		28.4%		25.2%
Type 0 soft wheat flour	60.1%	20.6%	50.1%	17.1%	45.1%	15.4%	40.1%	13.6%
*Biga*	6.0%	5.9%	6.0%	5.9%	6.0%	5.9%	6.0%	5.9%
Water	33.5%	35.1%	33.5%	35.1%	33.5%	35.1%	33.5%	35.1%
Baker’s yeast	0.4%	0.2%	0.4%	0.2%	0.4%	0.2%	0.4%	0.2%

SWB: bread produced with type 0 soft wheat flour; WWB: bread produced with whole wheat flour.

**Table 2:** Microbial viable counts of olive pomace samples.

Panel a					
		Total Mesophilic Aerobes	Presumptive Lactobacilli	Enterobacteriaceae	Eumycetes
f-OP	t_0_	5.00 ± 0.12 ^a^	2.96 ± 0.01 ^a^	<1.00	4.81 ± 0.15 ^d^
R-OP	t_1_	5.02 ± 0.04 ^a^	1.87 ± 0.04 ^b^	<1.00	5.69 ± 0.02 ^c^
	t_2_	4.07 ± 0.16 ^b^	1.65 ± 0.07 ^c^	<1.00	6.04 ± 0.07 ^b^
	t_3_	1.95 ± 0.07 ^c^	1.54 ± 0.09 ^c^	<1.00	6.24 ± 0.02 ^b^
	t_4_	1.83 ± 0.18 ^c^	1.86 ± 0.03 ^b^	<1.00	6.64 ± 0.00 ^a^
	t_5_	1.69 ± 0.12 ^c^	<1.00	<1.00	6.54 ± 0.01 ^a^
	t_6_	<1.00	<1.00	<1.00	6.54 ± 0.00 ^a^
**Panel b**					
		**Total Mesophilic Aerobes**	**Presumptive Lactobacilli**	**Enterobacteriaceae**	**Eumycetes**
f-OP	t_0_	5.00 ± 0.12 ^a^	2.96 ± 0.01 ^a^	<1.00	4.81 ± 0.15 ^a^
F-OP	t_1_	3.59 ± 0.03 ^b^	2.13 ± 0.07 ^b^	<1.00	3.33 ± 0.02 ^b^
	t_2_	3.51 ± 0.00 ^b^	2.06 ± 0.03 ^b^	<1.00	3.33 ± 0.01 ^b^
	t_3_	3.09 ± 0.01 ^c^	1.99 ± 0.02 ^b^	<1.00	3.27 ± 0.01 ^b^
	t_4_	2.74 ± 0.03 ^d^	1.98 ± 0.04 ^b^	<1.00	3.05 ± 0.05 ^bc^
	t_5_	2.87 ± 0.04 ^d^	1.81 ± 0.05 ^c^	<1.00	2.75 ± 0.21 ^c^
	t_6_	2.89 ± 0.06 ^cd^	2.08 ± 0.05 ^b^	<1.00	3.08 ± 0.01 ^bc^

f-OP: fresh olive pomace; R-OP: refrigerated olive pomace; F-OP: frozen olive pomace. t_0_: fresh olive pomace; t_1_, t_2_, t_3_, t_4_, t_5_, and t_6_ represent olive pomace samples after 1, 2, 3, 4, 5, and 6 months of storage, respectively. Results are expressed as mean Log cfu g^−1^ ± standard deviation. For each panel, the different letters in the same column indicate significant differences according to the Tukey–Kramer (HSD) test (*p* < 0.05).

**Table 3:** Total phenolic content determination and evaluation of the antioxidant capacity of olive pomace.

Panel a					
		TPC mg GAE g^−1^	DPPH mg TEAC g^−1^	ABTS mg TEAC g^−1^	FRAP mg TEAC g^−1^
f-OP	t_0_	7.18 ± 0.12 ^a^	16.21 ± 0.41 ^a^	15.36 ± 0.51 ^a^	15.95 ± 0.19 ^a^
R-OP	t_1_	7.35 ± 0.16 ^a^	16.15 ± 0.11 ^a^	14.69 ± 0.18 ^a^	16.48 ± 0.24 ^a^
	t_2_	6.80 ± 0.19 ^a^	15.47 ± 0.08 ^a^	14.78 ± 0.02 ^a^	14.67 ± 0.66 ^ab^
	t_3_	7.01 ± 0.12 ^a^	15.41 ± 0.32 ^a^	14.57 ± 0.25 ^a^	14.65 ± 0.35 ^ab^
	t_4_	7.40 ± 0.09 ^a^	16.47 ± 0.67 ^a^	16.49 ± 0.32 ^a^	15.71 ± 0.60 ^a^
	t_5_	6.67 ± 0.15 ^b^	14.87 ± 0.28 ^a^	14.42 ± 0.32 ^a^	14.34 ± 0.28 ^b^
	t_6_	7.39 ± 0.28 ^a^	16.23 ± 0.52 ^a^	15.86 ± 0.28 ^a^	15.36 ± 0.02 ^a^
**Panel b**					
		**TPC** **mg GAE g^−1^**	**DPPH** **mg TEAC g^−1^**	**ABTS** **mg TEAC g^−1^**	**FRAP** **mg TEAC g^−1^**
f-OP	t_0_	7.18 ± 0.12 ^a^	16.21 ± 0.41 ^a^	15.36 ± 0.51 ^a^	15.95 ± 0.19 ^a^
F-OP	t_1_	8.40 ± 0.59 ^a^	17.25 ± 0.44 ^a^	16.42 ± 0.27 ^ab^	18.16 ± 0.46 ^ab^
	t_2_	7.92 ± 0.40 ^a^	17.03 ± 0.45 ^a^	16.94 ± 0.04 ^ab^	18.56 ± 0.62 ^b^
	t_3_	7.38 ± 0.82 ^a^	16.27 ± 0.04 ^a^	17.13 ± 0.50 ^ab^	17.26 ± 0.31 ^a^
	t_4_	8.24 ± 0.31 ^a^	16.77 ± 0.22 ^a^	17.31 ± 0.58 ^ab^	18.88 ± 0.31 ^b^
	t_5_	7.98 ± 1.11 ^a,^*	16.80 ± 0.06 ^a,^*	18.33 ± 0.82 ^b,^*	18.99 ± 0.70 ^b,^*
	t_6_	9.05 ± 0.29 ^a,^*	17.41 ± 0.55 ^a,^*	15.80 ± 1.03 ^a^	20.20 ± 0.47 ^b,^*

TPC: Total phenolic content, DPPH radical-scavenging activity test; ABTS radical-scavenging activity assay; FRAP: ferric-ion-reducing power. f-OP: fresh olive pomace; R-OP: refrigerated olive pomace; F-OP: frozen olive pomace. t_0_: fresh olive pomace; t_1_, t_2_, t_3_, t_4_, t_5_, and t_6_ represent olive pomace samples after 1, 2, 3, 4, 5, and 6 months of storage, respectively. Results are expressed as mg GAE g^−1^ and mg TEAC g^−1^ on a wet basis; different letters indicate significant differences among samples from the two groups (refrigerated and frozen samples; one-way ANOVA test); stars indicate statistical differences among samples in comparison to the corresponding samples of the other sample group (comparison between refrigerated versus frozen samples analyzed at the same storing time; *t*-test).

**Table 4:** Viable counts of experimental bread doughs.

	% OP	Presumptive Lactobacilli				Eumycetes			
		SWD		WWD		SWD		WWD	
		BL	AL	BL	AL	BL	AL	BL	AL
t_0_-f	0%	1.83 ± 0.18 ^b,A^	2.02 ± 0.09 ^a,A^	1.72 ± 0.17 ^b,A^	1.39 ± 0.12 ^b,A^	6.40 ± 0.00 ^b,A^	6.30 ± 0.06 ^ab,A^	6.50 ± 0.01 ^a,A^	6.30 ± 0.00 ^b,B^
	10%	2.60 ± 0.18 ^a,A^	1.98 ± 0.71 ^a,A^	2.40 ± 0.02 ^ab,A^	2.55 ± 0.04 ^a,A^	6.59 ± 0.02 ^a,A^	6.37 ± 0.04 ^a,B^	6.45 ± 0.03 ^a,A^	6.24 ± 0.05 ^b,B^
	15%	2.76 ± 0.11 ^a,A^	2.94 ± 0.12 ^a,A^	2.69 ± 0.51 ^ab,A^	2.88 ± 0.18 ^a,A^	6.57 ± 0.00 ^ab,A^	6.20 ± 0.01 ^b,B^	6.54 ± 0.10 ^a,A^	6.78 ± 0.03 ^a,A^
	20%	2.69 ± 0.04 ^a,A^	3.04 ± 0.33 ^a,A^	3.04 ± 0.10 ^a,A^	3.02 ± 0.13 ^a,A^	6.55 ± 0.09 ^ab,A^	6.21 ± 0.04 ^ab,B^	6.59 ± 0.01 ^a,A^	5.96 ± 0.01 ^c,B^
t_6_-R	0%	1.48 ± 0.40 ^b,A^	1.50 ± 0.24 ^c,A^	1.86 ± 0.45 ^b,A^	2.32 ± 0.22 ^a,A^	6.92 ± 0.10 ^b,A^	7.05 ± 0.08 ^a,A^	6.26 ± 0.10 ^b,A^	6.72 ± 0.03 ^c,A^
	10%	3.21 ± 0.24 ^a,A^	3.06 ± 0.33 ^b,A^	2.96 ± 0.36 ^ab,A^	2.73 ± 0.84 ^a,A^	7.28 ± 0.13 ^a,A^	7.08 ± 0.10 ^a,A^	7.14 ± 0.04 ^a,A^	7.00 ± 0.01 ^a,B^
	15%	3.31 ± 0.25 ^a,A^	3.32 ± 0.32 ^ab,A^	3.48 ± 0.92 ^a,A^	3.33 ± 1.11 ^a,A^	7.27 ± 0.17 ^a,A^	7.10 ± 0.12 ^a,A^	7.03 ± 0.02 ^a,A^	7.05 ± 0.01 ^a,A^
	20%	3.48 ± 0.13 ^a,A^	3.66 ± 0.20 ^a,A^	3.69 ± 0.66 ^a,A^	3.34 ± 0.77 ^a,A^	7.35 ± 0.11 ^a,A^	7.17 ± 0.11 ^a,A^	7.09 ± 0.01 ^a,A^	6.81 ± 0.06 ^b,B^
t_6_-F	0%	1.48 ± 0.40 ^b,A^	1.50 ± 0.24 ^a,A^	1.86 ± 0.45 ^a,A^	2.32 ± 0.22 ^a,A^	6.92 ± 0.10 ^b,A^	7.05 ± 0.08 ^ab,A^	6.26 ± 0.10 ^d,B^	6.72 ± 0.03 ^c,A^
	10%	1.86 ± 0.28 ^ab,A^	1.56 ± 0.40 ^a,A^	1.76 ± 0.15 ^a,A^	1.84 ± 0.17 ^a,A^	7.29 ± 0.14 ^a,A^	7.29 ± 0.12 ^a,A^	6.38 ± 0.02 ^c,A^	6.23 ± 0.06 ^d,B^
	15%	2.31 ± 0.39 ^a,A^	1.51 ± 0.37 ^a,B^	2.08 ± 0.27 ^a,A^	1.75 ± 0.50 ^a,A^	7.41 ± 0.14 ^a,A^	7.15 ± 0.12 ^ab,B^	7.10 ± 0.01 ^a,A^	6.91 ± 0.04 ^b,B^
	20%	1.15 ± 0.30 ^b,A^	1.80 ± 0.55 ^a,A^	2.60 ± 0.96 ^a,A^	1.83 ± 1.59 ^a,A^	7.37 ± 0.14 ^a,A^	7.23 ± 0.07 ^ab,A^	6.83 ± 0.01 ^b,B^	7.07 ± 0.01 ^a,A^

% OP: percentage of olive pomace added to dough; SWD: dough produced with soft wheat flour type 0; WWD: dough produced with whole wheat flour; BL: dough before leavening; AL: dough after leavening. t_0_-f: dough made using freshly sampled OP; t_6_-R: dough made using olive pomace after 6 months of refrigeration; t_6_-F: dough made using olive pomace after 6 months of freezing. Results are expressed as mean Log cfu g^−1^ ± standard deviation. For each parameter, within each type of olive pomace storage technique, and considering the flour type used, means followed by different letters within each column (lowercase) and each row (capital letters) indicate significant differences (*p* < 0.05).

**Table 5:** Specific volume and hardness of experimental bread samples.

	%OP	Specific Volume (cm^3^ g^−1^)		Hardness (N)	
		SWB	WWB	SWB	WWB
t_0_-f	0%	22.14 ± 0.01 ^a,A^	20.81 ± 0.05 ^a,B^	22.75 ± 3.17 ^a,A^	25.86 ± 1.73 ^a,A^
	10%	21.23 ± 1.06 ^a,A^	20.76 ± 0.03 ^a,A^	10.83 ± 1.20 ^c,B^	21.57 ± 2.31 ^c,A^
	15%	20.91 ± 1.30 ^a,A^	21.93 ± 0.72 ^a,A^	11.74 ± 1.22 ^c,B^	26.29 ± 1.73 ^b,A^
	20%	22.56 ± 0.20 ^a,A^	21.95 ± 0.07 ^a,A^	18.75 ± 0.47 ^b,B^	23.00 ± 2.01 ^b,A^
t_6_-R	0%	22.65 ± 0.82 ^a,A^	19.21 ± 1.20 ^a,B^	22.40 ± 2.22 ^a,B^	26.90 ± 0.95 ^a,A^
	10%	20.40 ± 1.04 ^a,A^	20.11 ± 0.77 ^a,A^	8.09 ± 0.94 ^b,B^	16.66 ± 1.95 ^b,A^
	15%	20.31 ± 1.35 ^a,A^	21.48 ± 1.01 ^a,A^	9.73 ± 1.33 ^b,B^	24.30 ± 1.87 ^a,A^
	20%	21.68 ± 0.76 ^a,A^	21.15 ± 2.59 ^a,A^	13.34 ± 1.52 ^b,B^	24.01 ± 1.51 ^ab,A^
t_6_-F	0%	22.65 ± 0.82 ^a,A^	19.21 ± 1.20 ^a,B^	22.40 ± 2.22 ^b,B^	26.90 ± 0.94 ^b,A^
	10%	21.07 ± 0.62 ^a,A^	19.41 ± 2.34 ^a,A^	19.26 ± 2.74 ^b,B^	25.59 ± 4.91 ^b,A^
	15%	21.07 ± 1.09 ^a,A^	21.67 ± 1.48 ^a,A^	28.21 ± 1.18 ^a,B^	31.26 ± 2.47 ^a,A^
	20%	22.56 ± 0.95 ^a,A^	20.80 ± 0.43 ^a,B^	29.12 ± 3.73 ^a,B^	33.72 ± 1.27 ^a,A^

% OP: percentage of added olive pomace. SWB: bread produced with soft wheat flour type 0; WWB: bread produced with whole wheat flour. t_0_-f: bread made using freshly sampled olive pomace; t_6_-R: bread made using olive pomace after 6 months of refrigeration; t_6_-F: bread made using olive pomace after 6 months of freezing. For each parameter, for each type of olive pomace storage technique, and considering the flour type used, means followed by different letters within each column (lowercase) and each row (capital letters) indicate significant differences (*p* < 0.05).

**Table 6:** Total phenolic content, antioxidant capacity (AOC) as determined using the FOLIN, DPPH, ABTS, and FRAP assays, and dietary fiber content of bread samples.

	%OP	TPC (mg GAE g^−1^)		DPPH (mg TEAC g^−1^)		ABTS (mg TEAC g^−1^)		FRAP (mg TEAC g^−1^)		Dietary Fiber (g 100 g^−1^)	
		SWB	WWB	SWB	WWB	SWB	WWB	SWB	WWB	SWB	WWB
t_0_-f	0%	0.27 ± 0.01 ^a, A^	0.29± 0.061 ^a,A^	0.19 ± 0.03 ^a,A^	0.28 ± 0.10 ^a,A^	0.32 ± 0.01 ^a,A^	0.56 ± 0.13 ^a,A^	0.14 ± 0.01 ^a,A^	0.25 ± 0.47 ^a,A^	3.27 ± 0.08 ^c,B^	6.09 ± 0.83 ^a,A^
	10%	0.70 ± 0.08 ^b,A^	0.68 ± 0.05 ^b,A^	1.26 ± 0.17 ^bc,A^	1.29 ± 0.00 ^b,A^	1.23 ± 0.22 ^b,A^	1.41 ± 0.04 ^b,A^	1.15 ± 0.10 ^b,A^	1.23 ± 0.04 ^b,A^	4.43 ± 0.52 ^b,A^	6.83 ± 0.59 ^a,A^
	15%	0.84 ± 0.09 ^bc,A^	0.90 ± 0.10 ^bc,A^	1.67 ± 0.09 ^bc,A^	1.78 ± 0.05 ^bc,A^	1.55 ± 0.01 ^bc,A^	1.73 ± 0.10 ^b,A^	1.63 ± 0.07 ^b,A^	1.65 ± 0.05 ^b,A^	5.49 ± 0.09 ^ab,A^	7.00 ± 0.69 ^a,A^
	20%	0.91 ± 0.05 ^c,A^	0.75 ± 0.11 ^bc,A^	1.90 ± 0.18 ^c,A^	1.12 ± 0.00 ^b,A^	1.76 ± 0.05 ^c,A^	1.24 ± 0.04 ^b,B^	1.76 ± 0.16 ^b,A^	1.11 ± 0.01 ^b,B^	6.57 ± 0.09 ^a,A^	8.71 ± 0.83 ^a,A^
t_6_-R	0%	0.19 ± 0.01 ^a,A^	0.37 ± 0.04 ^a,B^	0.25 ± 0.04 ^a,A^	0.33 ± 0.03 ^a,A^	0.77 ± 0.11 ^a,A^	0.97 ± 0.05 ^a,A^	0.21 ± 0.00 ^a,A^	0.31 ± 0.01 ^a,A^	3.36 ± 0.20 ^b,B^	5.20 ± 0.44 ^a,A^
	10%	1.08 ± 0.28 ^b,A^	0.99 ± 0.05 ^b,A^	0.89 ± 0.21 ^b,A^	0.82 ± 0.19 ^b,A^	1.93 ± 0.49 ^b,A^	1.82 ± 0.04 ^b,A^	1.38 ± 0.49 ^b,A^	1.21 ±0.30 ^b,A^	4.83 ± 0.60 ^ab,A^	6.58 ± 1.27 ^a,A^
	15%	1.46 ± 0.33 ^b,A^	1.20 ± 0.13 ^b,A^	1.21 ± 0.21 ^b,A^	0.97 ± 0.01 ^b,A^	2.57 ± 0.50 ^c,A^	2.15 ± 0.04 ^bc,A^	2.02 ± 0.59 ^bc,A^	1.45 ±0.03 ^b,B^	4.87 ± 1.03 ^ab,A^	6.65 ± 0.88 ^a,A^
	20%	1.70 ± 0.20 ^b,A^	1.61 ± 0.52 ^b,A^	1.36 ± 0.27 ^b,A^	1.34 ± 0.33 ^b,A^	2.81 ± 0.45 ^c,A^	2.64 ± 0.58 ^c,A^	2.35 ± 0.51 ^c,A^	2.14 ±0.81 ^c,A^	6.44 ± 0.59 ^a,A^	7.34 ± 0.44 ^a,A^
t_6_-F	0%	0.19 ± 0.01 ^a,A^	0.37 ± 0.04 ^a,B^	0.25 ± 0.04 ^a,A^	0.33 ± 0.03 ^a,A^	0.77 ± 0.11 ^a,A^	0.97 ± 0.05 ^a,A^	0.21 ± 0.00 ^a,A^	0.31 ± 0.01 ^a,A^	3.36 ± 0.20 ^b,B^	5.20 ± 0.44 ^a,A^
	10%	0.91 ± 0.18 ^b,A^	0.95 ± 0.05 ^b,A^	0.62 ± 0.08 ^b,A^	0.66 ± 0.16 ^b,A^	1.56 ± 0.18 ^b,A^	1.64 ± 0.71 ^b,A^	1.01 ± 0.22 ^b,A^	0.99 ± 0.21 ^b,A^	4.32 ± 0.72 ^ab,A^	5.32 ± 0.71 ^a,A^
	15%	1.01 ± 0.33 ^b,A^	0.72 ± 0.14 ^b,A^	1.01 ± 0.38 ^b,A^	0.82 ± 0.03 ^b,A^	2.11 ± 0.72 ^c,A^	1.58 ± 0.13 ^b,A^	1.53 ± 0.73 ^b,A^	1.13 ± 0.10 ^b,A^	5.65 ± 0.83 ^a,A^	5.97 ± 1.35 ^a,A^
	20%	1.39 ± 0.60 ^b,A^	1.22 ± 0.13 ^b,A^	1.07 ± 0.39 ^b,A^	1.27 ± 0.26 ^b,A^	2.36 ± 0.40 ^c,A^	2.30 ± 0.01 ^c,A^	1.85 ± 0.64 ^c,A^	1.69 ± 0.24 ^c,A^	6.21 ± 0.67 ^a,A^	6.75 ± 0.50 ^a,A^

% OP: percentage of olive pomace added to bread; TPC: total phenolic content; DPPH radical-scavenging activity test; ABTS radical-scavenging activity assay; FRAP: ferric-ion-reducing power. SWB: bread produced with soft wheat flour type 0; WWB: bread produced with whole wheat flour. t_0_-f: bread made using freshly sampled olive pomace; t_6_-R: bread made using olive pomace after 6 months of refrigeration; t_6_-F: bread made olive pomace after 6 months of freezing. Results are expressed as mg GAE g^−1^ and mM TEAC g^−1^ on a wet basis. For each parameter, within each type of olive pomace storage technique and considering the flour type used, means followed by different letters within each column (lowercase) and each row (capital letters) indicate significant differences (*p* < 0.05).

**Table 7:** Volatile organic compounds (VOCs) identified in bread samples made with soft wheat flour (SWB) and with different concentration of olive pomace (0%, 10%, 15%, or 20%) and stored under different conditions (f, fresh; F, frozen; R, refrigerated).

RI	Compounds	t_0_-f 0%	t_0_-f 10%	t_0_-f 15%	t_0_-f 20%	t_6_-(R or F) 0%	t_6_-R 10%	t_6_-R 15%	t_6_-R 20%	t_6_-F 10%	t_6_-F 15%	t_6_-F 20%
	**Terpenoids**											
1084	beta pinene	1.90 ± 0.03 ^a^	3.00 ± 0.05 ^c^	4.10 ± 0.05 ^d^	5.46 ± 0.51 ^e^	1.95 ± 0.04 ^a^	1.43 ± 0.26 ^b^	3.19 ± 0.53 ^c^	3.17 ± 0.12 ^c^	2.98 ± 0.20 ^c^	3.48 ± 0.17 ^c^	4.44 ± 0.23 ^d^
1191	limonene	0.20 ± 0.01 ^b^	0.30 ± 0.02 ^c^	0.35 ± 0.03 ^c^	0.50 ± 0.02 ^d^	0.26 ± 0.01 ^a^	0.24 ± 0.05 ^a^	0.28 ± 0.01 ^a^	0.34 ± 0.05 ^c^	0.30 ± 0.08 ^c^	0.32 ± 0.05 ^c^	0.49 ± 0.08 ^d^
1480	Copaene	Nd	2.14 ± 0.00 ^d^	2.50 ± 0.02 ^e^	2.84 ± 0.01 ^f^	Nd	0.87 ± 0.02 ^a^	1.04 ± 0.06 ^b^	1.33 ± 0.19 ^c^	1.20 ± 0.04 ^c^	1.26 ± 0.29 ^c^	1.40 ± 0.05 ^c^
	**Ketones**											
812	2-Butanone	Nd	0.20 ± 0.00 ^d^	0.21 ± 0.01 ^d^	0.37 ± 0.01 ^b^	Nd	0.60 ± 0.12 ^a^	0.49 ± 0.03 ^a^	0.37 ± 0.02 ^b^	Nd	Nd	0.16 ± 0.01 ^c^
938	2-pentanone	Nd	5.61 ± 0.14 ^a^	6.56 ± 0.52 ^a^	6.51 ± 0.42 ^a^	Nd	Nd	6.01 ± 1.06 ^a^	5.73 ± 0.34 ^a^	Nd	Nd	Nd
1065	2,3-pentanedione	0.22 ± 0.01 ^b^	2.18 ± 0.09 ^f^	5.88 ± 0.51 ^g^	7.48 ± 0.51 ^h^	0.26 ± 0.01 ^a^	0.92 ± 0.17 ^c^	0.79 ± 0.21 ^c^	0.73 ± 0.12 ^c^	0.54 ± 0.02 ^d^	0.22 ± 0.01 ^a^	0.15 ± 0.03 ^e^
1198	2-heptanone	0.48 ± 0.15 ^a^	0.48 ± 0.04 ^a^	1.10 ± 0.09 ^b^	1.85 ± 0.04 ^c^	0.52 ± 0.06 ^a^	Nd	Nd	Nd	Nd	Nd	Nd
1283	2-octanone	0.16 ± 0.01 ^b^	Nd	Nd	Nd	0.11 ± 0.01 ^a^	0.35 ± 0.04 ^c^	0.47 ± 0.08 ^d^	0.78 ± 0.03 ^e^	Nd	0.22 ± 0.02 ^f^	0.33 ± 0.01 ^c^
1281	Acetoin	0.77 ± 0.03 ^b^	2.18 ± 0.01 ^d^	2.57 ± 0.04 ^d^	2.75 ± 0.01 ^d^	3.16 ± 0.59 ^a^	4.01 ± 0.84 ^c^	2.21 ± 0.49 ^d^	1.85 ± 0.47 ^ed^	1.34 ± 0.08 ^e^	2.04 ± 0.22 ^d^	2.10 ± 0.35 ^d^
1303	Acetol	0.50 ± 0.01 ^b^	1.40 ± 0.00 ^c^	0.99 ± 0.00 ^c^	0.12 ± 0.03 ^e^	0.66 ± 0.03 ^a^	1.38 ± 0.19 ^c^	1.08 ± 0.24 ^c^	0.90 ± 0.09 ^c^	0.57 ± 0.04 ^d^	0.42 ± 0.09 ^b^	0.40 ± 0.08 ^b^
	**Aldehydes**											
820	2-methylbutanal	11.96 ± 0.63 ^b^	16.28 ± 0.49 ^d^	10.22 ± 0.22 ^a^	5.52 ± 0.12 ^c^	10.18 ± 0.06 ^a^	10.38 ± 0.78 ^a^	5.71 ± 0.42 ^c^	5.11 ± 1.10 ^c^	10.89 ± 0.12 ^b^	9.68 ± 0.14 ^a^	5.28 ± 0.13 ^c^
825	3-methylbutanal	14.07 ± 0.59 ^b^	30.84 ± 0.46 ^g^	21.18 ± 0.32 ^h^	13.62 ± 0.17 ^b^	10.63 ± 0.08 ^a^	16.75 ± 1.01 ^c^	10.54 ± 0.43 ^a^	10.25 ± 1.22 ^d^	10.99 ± 0.18 ^ad^	9.21 ± 0.15 ^e^	7.25 ± 0.24 ^f^
1078	Hexanal	7.78 ± 0.50 ^b^	6.46 ± 0.19 ^f^	8.99 ± 0.47 ^g^	9.56 ± 0.20 ^h^	5.62 ± 0.43 ^a^	3.81 ± 0.46 ^c^	2.55 ± 0.19 ^d^	2.46 ± 0.13 ^d^	1.17 ± 0.22 ^e^	1.58 ± 0.05 ^e^	1.68 ± 0.32 ^e^
1325	2-heptenal	0.10 ± 0.00 ^a^	1.24 ± 0.08 ^e^	1.07 ± 0.05 ^b^	0.92 ± 0.02 ^b^	Nd	1.07 ± 0.13 ^b^	0.90 ± 0.05 ^c^	1.19 ± 0.20 ^b^	0.54 ± 0.14 ^d^	0.45 ± 0.09 ^d^	0.63 ± 0.07 ^d^
1522	Benzaldehyde	1.16 ± 0.06 ^b^	9.76 ± 0.19 ^e^	12.98 ± 0.75 ^f^	15.90 ± 0.10 ^g^	0.97 ± 0.04 ^a^	3.91 ± 0.88 ^c^	4.46 ± 0.33 ^c^	4.73 ± 0.92 ^c^	4.28 ± 0.72 ^c^	4.97 ± 0.23 ^c^	7.72 ± 1.24 ^d^
1640	Benzeneacetaldehyde	Nd	0.87 ± 0.09 ^c^	1.18 ± 0.03 ^d^	1.23 ± 0.03 ^d^	Nd	0.23 ± 0.05 ^a^	0.52 ± 0.11 ^b^	0.60 ± 0.10 ^b^	Nd	0.84 ± 0.10 ^c^	0.78 ± 0.12 ^c^
	**Esters and acetates**											
1433	Ethyl octanoate	0.30 ± 0.01 ^a^	0.30 ± 0.00 ^c^	0.32 ± 0.00 ^d^	0.86 ± 0.04 ^e^	Nd	0.25 ± 0.05 ^a^	0.30 ± 0.02 ^a^	0.74 ± 0.17 ^b^	0.50 ± 0.11 ^b^	0.61 ± 0.05 ^b^	0.64 ± 0.10 ^b^
1660	Ethyl benzoate	Nd	0.30 ± 0.01 ^a^	0.31 ± 0.02 ^a^	0.28 ± 0.01 ^a^	Nd	0.33 ± 0.09 ^a^	0.87 ± 0.10 ^b^	0.88 ± 0.03 ^b^	0.57 ± 0.03 ^c^	0.55 ± 0.04 ^c^	0.47 ± 0.07 ^c^
801	Ethyl acetate	1.96 ± 0.01 ^a^	2.60 ± 0.00 ^e^	3.75 ± 0.04 ^b^	4.01 ± 0.05 ^d^	2.18 ± 0.35 ^a^	1.86 ± 0.13 ^a^	3.64 ± 0.67 ^b^	5.75 ± 0.48 ^c^	3.40 ± 0.31 ^b^	3.88 ± 0.19 ^b^	4.18 ± 0.24 ^d^
	**Alcohols**											
888	Ethanol	134.31 ± 8.04 ^b^	86.71 ± 7.33 ^f^	128.4 ± 3.27 ^g^	120.2 ± 0.67 ^h^	105.61 ± 5.58 ^a^	101.1 ± 20.77 ^a^	183.8 ± 37.12 ^c^	174.2 ± 9.15 ^c^	254.3 ± 12.73 ^d^	225.7 ± 36.31 ^d^	212.3 ± 25.10 ^e^
1211	Isoamylalcohol	17.53 ± 0.12 ^a^	26.90 ± 3.63 ^a^	30.08 ± 0.28 ^b^	42.26 ± 0.17 ^b^	16.57 ± 3.19 ^a^	17.95 ± 4.31 ^a^	20.86 ± 0.86 ^a^	20.15 ± 3.22 ^a^	21.12 ± 4.88 ^a^	19.09 ± 4.92 ^a^	19.33 ± 2.98 ^a^
1108	Isobutanol	2.29 ± 0.07 ^b^	2.50 ± 0.01 ^b^	3.40 ± 0.02 ^a^	9.46 ± 0.44 ^c^	3.50 ± 0.47 ^a^	2.85 ± 0.51 ^b^	2.47 ± 0.25 ^b^	2.38 ± 0.13 ^b^	3.18 ± 0.31 ^a^	3.58 ± 0.31 ^a^	3.94 ± 1.18 ^a^
1355	1-Hexanol	1.72 ± 0.12 ^a^	7.37 ± 0.04 ^d^	8.10 ± 0.26 ^e^	15.03 ± 0.90 ^f^	1.57 ± 0.43 ^a^	2.83 ± 0.53 ^b^	2.99 ± 0.80 ^b^	2.50 ± 0.20 ^b^	3.94 ± 0.85 ^c^	4.38 ± 0.28 ^c^	5.21 ± 0.78 ^c^
1383	3-hexen-1-ol	Nd	Nd	1.10 ± 0.10 ^c^	1.78 ± 0.02 ^d^	Nd	Nd	Nd	0.39 ± 0.02 ^a^	Nd	0.82 ± 0.02 ^b^	0.90 ± 0.10 ^b^
1905	Benzeneethanol	1.74 ± 0.01 ^b^	1.21 ± 0.01 ^d^	1.31 ± 0.03 ^e^	1.51 ± 0.00 ^f^	2.14 ± 0.61 ^a^	2.31 ± 0.27 ^a^	2.49 ± 0.18 ^a^	3.40 ± 0.2 ^c^	1.69 ± 0.26 ^b^	1.83 ± 0.26 ^b^	2.09 ± 0.04 ^a^
	**Pyrazines**											
1268	Methylpyrazine	5.00 ± 0.11 ^b^	5.50 ± 0.01 ^b^	7.47 ± 0.42 ^c^	9.86 ± 0.01 ^f^	4.50 ± 0.18 ^a^	5.85 ± 1.03 ^b^	8.72 ± 1.05 ^c^	10.27 ± 1.35 ^d^	3.26 ± 0.19 ^e^	4.81 ± 0.55 ^a^	7.61 ± 1.18 ^c^
1325	2,5-dimethyl pyrazine	0.71 ± 0.02 ^a^	0.75 ± 0.01 ^a^	0.71 ± 0.04 ^a^	1.50 ± 0.01 ^e^	0.65 ± 0.11 ^a^	0.93 ± 0.12 ^b^	1.21 ± 0.09 ^c^	2.96 ± 0.57 ^d^	0.86 ± 0.16 ^b^	0.81 ± 0.04 ^b^	1.55 ± 0.07 ^e^
1349	2,3-dimethylpyrazine	1.11 ± 0.14 ^a^	Nd	Nd	Nd	1.00 ± 0.12 ^a^	1.45 ± 0.15 ^b^	0.45 ± 0.02 ^c^	0.29 ± 0.03 ^d^	0.71 ± 0.04 ^e^	0.19 ± 0.01 ^f^	0.51 ± 0.02 ^g^
1386	2-ethyl-6-methylpyrazine	0.78 ± 0.12 ^b^	1.98 ± 0.06 ^g^	2.15 ± 0.03 ^f^	2.35 ± 0.01 ^h^	0.97 ± 0.07 ^a^	0.98 ± 0.13 ^a^	1.38 ± 0.17 ^c^	2.60 ± 0.13 ^d^	1.11 ± 0.01 ^e^	1.52 ± 0.02 ^c^	2.12 ± 0.03 ^f^
1391	2-ethyl-5-methylpyrazine	1.55 ± 0.09 ^a^	1.77 ± 0.04 ^e^	2.01 ± 0.02 ^f^	2.65 ± 0.02 ^g^	1.65 ± 0.08 ^a^	1.17 ± 0.12 ^b^	1.58 ± 0.10 ^a^	3.18 ± 0.25 ^c^	0.91 ± 0.16 ^b^	1.20 ± 0.04 ^b^	1.35 ± 0.11 ^d^
	**Acids**											
1447	Acetic acid	11.83 ± 0.47 ^b^	16.41 ± 0.25 ^a^	19.83 ± 0.43 ^c^	20.02 ± 0.53 ^c^	13.77 ± 0.64 ^a^	21.44 ± 1.76 ^c^	23.16 ± 2.46 ^c^	27.76 ± 0.45	15.40 ± 2.06 ^a^	20.39 ± 2.73 ^c^	22.57 ± 2.89 ^c^
1628	Butanoic acid	0.54 ± 0.00 ^b^	0.85 ± 0.02 ^c^	1.03 ± 0.03 ^d^	1.15 ± 0.02 ^e^	0.62 ± 0.04 ^a^	0.76 ± 0.06 ^c^	0.94 ± 0.13 ^d^	0.98 ± 0.11 ^d^	0.91 ± 0.22 ^d^	1.23 ± 0.15 ^e^	1.23 ± 0.18 ^e^
1840	Hexanoic acid	Nd	0.45 ± 0.01 ^c^	0.54 ± 0.01 ^d^	0.59 ± 0.00 ^d^	0.21 ± 0.01 ^a^	0.35 ± 0.04 ^b^	0.48 ± 0.08 ^c^	0.58 ± 0.08 ^d^	0.41 ± 0.03 ^c^	0.40 ± 0.01 ^c^	0.50 ± 0.09 ^c^
2058	Octanoic acid	0.25 ± 0.01 ^b^	0.22 ± 0.00 ^a^	0.26 ± 0.06 ^b^	0.52 ± 0.10 ^f^	0.21 ± 0.04 ^a^	0.37 ± 0.06 ^c^	0.20 ± 0.00 ^a^	0.14 ± 0.01 ^d^	0.09 ± 0.00 ^e^	0.14 ± 0.01 ^d^	0.16 ± 0.01 ^d^
2281	Decanoic acid	0.30 ± 0.01 ^b^	0.34 ± 0.03 ^a^	0.46 ± 0.04 ^f^	0.70 ± 0.04 ^g^	0.35 ± 0.02 ^a^	0.10 ± 0.01 ^c^	0.32 ± 0.02 ^ab^	0.94 ± 0.17 ^d^	0.23 ± 0.03 ^e^	0.25 ± 0.04 ^e^	0.35 ± 0.04 ^a^
	**Furans and pyrans**											
1223	2-pentylfuran	4.02 ± 0.03 ^b^	3.78 ± 0.10 ^f^	1.97 ± 0.04 ^g^	1.91 ± 0.01 ^h^	3.50 ± 0.02 ^a^	Nd	0.49 ± 0.05 ^c^	0.81 ± 0.03 ^d^	0.49 ± 0.06 ^c^	0.45 ± 0.05 ^c^	0.40 ± 0.01 ^e^
1462	2-furfural	2.10 ± 0.02 ^b^	5.08 ± 0.06 ^g^	5.24 ± 0.34 ^g^	5.50 ± 0.31 ^d^	2.64 ± 0.31 ^a^	9.65 ± 1.87 ^c^	7.70 ± 1.49 ^d^	6.35 ± 0.65 ^d^	3.37 ± 0.49 ^e^	4.05 ± 0.21 ^f^	2.04 ± 0.91 ^b^
1658	2-Furanmethanol	1.61 ± 0.06 ^a^	8.43 ± 0.47 ^c^	7.59 ± 0.24 ^g^	7.38 ± 0.21 ^g^	2.10 ± 0.62 ^a^	12.11 ± 2.41 ^b^	8.61 ± 0.57 ^c^	3.88 ± 0.25 ^d^	5.89 ± 0.92 ^e^	3.36 ± 0.17 ^f^	2.27 ± 0.58 ^a^
1503	2-acetylfuran	0.28 ± 0.00 ^a^	1.07 ± 0.01 ^c^	0.79 ± 0.05 ^f^	0.73 ± 0.03 ^f^	0.27 ± 0.06 ^a^	1.27 ± 0.17 ^b^	1.14 ± 0.21 ^c^	0.49 ± 0.06 ^d^	0.64 ± 0.06 ^e^	0.59 ± 0.08 ^de^	0.55 ± 0.11 ^d^
2501	5-(hydroxymethyl)-2-furfural (HMF)	0.31 ± 0.00 ^b^	0.46 ± 0.02 ^a^	0.51 ± 0.03 ^a^	0.62 ± 0.03 ^d^	0.45 ± 0.12 ^a^	0.84 ± 0.15 ^c^	0.52 ± 0.07 ^a^	0.41 ± 0.06 ^a^	0.45 ± 0.03 ^a^	0.52 ± 0.01 ^a^	0.44 ± 0.06 ^a^
	**Phenols**											
2203	Vinylguaiacol	Nd	0.30 ± 0.05 ^c^	0.35 ± 0.04 ^c^	0.42 ± 0.02 ^d^	Nd	0.19 ± 0.01 ^a^	0.18 ± 0.04 ^ab^	0.13 ± 0.03 ^b^	0.24 ± 0.04 ^c^	0.24 ± 0.05 ^c^	0.31 ± 0.05 ^c^
1862	Guaiacol	Nd	0.48 ± 0.02 ^c^	0.51 ± 0.01 ^c^	0.60 ± 0.02 ^d^	Nd	0.32 ± 0.07 ^a^	0.36 ± 0.01 ^a^	0.42 ± 0.04 ^b^	0.32 ± 0.07 ^a^	0.40 ± 0.03 ^b^	0.41 ± 0.04 ^b^

RI: retention index. RIs were calculated using the van Den Dool and Kratz formula. Calculated RIs were compared using the online NIST database (http://webbook.nist.gov/chemistry/; accessed on 1 June 2023) for a high polar column for InnoWAX or similar stationary phases. All the compounds were identified by the matching RI and MS. The results are expressed as RAP ± SD; relative peak area (area peak compound/area peak internal standard) × 100 ± standard deviation. Values labeled with different lowercase letters in the same row are significantly different (*p* < 0.05). Nd: not detected.

**Table 8:** Volatile organic compounds (VOCs) identified in breads made with whole wheat flour (WWB) and with different concentrations of olive pomace (0%, 10%, 15%, or 20%) and stored under different conditions (f, fresh; F, frozen; R, refrigerated).

RI	Compounds	t_0_-f 0%	t_0_-f 10%	t_0_-f 15%	t_0_-f 20%	t_6_-(R or F) 0%	t_6_-R 10%	t_6_-R 15%	t_6_-R 20%	t_6_-F 10%	t_6_-F 15%	t_6_-F 20%
	**Terpenoids**											
1084	Beta pinene	3.23 ± 0.65 ^a^	4.60 ± 0.50 ^a^	4.80 ± 0.60 ^a^	6.10 ± 0.50 ^b^	3.50 ± 1.0 ^a^	4.10 ± 0.36 ^a^	4.50 ± 0.73 ^a^	4.60 ± 0.47 ^a^	4.48 ± 0.58 ^a^	4.85 ± 0.98 ^c^	2.98 ± 0.15 ^a^
1191	Limonene	0.55 ± 0.04 ^a^	0.57 ± 0.05 ^a^	0.95 ± 0.01 ^d^	2.44 ± 0.15 ^e^	0.60 ± 0.05 ^a^	0.47 ± 0.01 ^b^	0.47 ± 0.02 ^b^	0.60 ± 0.02 ^a^	0.60 ± 0.03 ^a^	0.74 ± 0.10 ^a^	1.12 ± 0.01 ^c^
1480	Copaene	Nd	1.57 ± 0.03 ^c^	2.94 ± 0.06 ^d^	3.18 ± 0.02 ^e^	Nd	0.95 ± 0.05 ^a^	1.14 ± 0.28 ^a,b^	1.23 ± 0.21 ^b^	0.85 ± 0.01 ^c,b^	0.87 ± 0.08 ^c,b^	1.04 ± 0.04 ^b^
	**Ketones**											
812	2-Butanone	0.33 ± 0.00 ^a^	0.31 ± 0.00 ^a^	0.36 ± 0.03 ^a^	0.85 ± 0.03 ^e^	0.34 ± 0.02 ^a^	0.21 ± 0.04 ^b^	0.39 ± 0.02 ^c^	0.57 ± 0.09 ^d^	0.19 ± 0.04 ^b^	0.25 ± 0.03 ^b^	0.36 ± 0.03 ^a^
938	2-pentanone	3.84 ± 0.36 ^b^	4.78 ± 0.15 ^e^	4.91 ± 0.05 ^e^	7.20 ± 0.40 ^f^	3.10 ± 0.05 ^a^	2.04 ± 0.20 ^c^	2.47 ± 0.20 ^d^	2.54 ± 0.05 ^d^	2.32 ± 0.23 ^c,d^	2.80 ± 0.30 ^d^	2.96 ± 0.59 ^d^
1065	2,3-pentanedione	0.40 ± 0.08 ^a^	1.10 ± 0.03 ^d^	2.08 ± 0.03 ^e^	2.20 ± 0.05 ^f^	0.38 ± 0.02 ^a^	0.31 ± 0.03 ^a^	0.47 ± 0.05 ^a^	0.72 ± 0.04 ^b^	0.23 ± 0.02 ^c^	0.32 ± 0.02 ^a^	0.67 ± 0.02 ^b^
1198	2-heptanone	0.70 ± 0.01 ^a^	Nd	Nd	Nd	0.75 ± 0.07 ^a^	Nd	Nd	Nd	Nd	Nd	Nd
1283	2-octanone	1.10 ± 0.05 ^a^	0.95 ± 0.05 ^c^	1.10 ± 0.06 ^a^	1.45 ± 0.02 ^e^	1.18 ± 0.27 ^a^	0.62 ± 0.12 ^b^	0.85 ± 0.05 ^c^	0.90 ± 0.05 ^c^	0.87 ± 0.03 ^c^	1.10 ± 0.05 ^a^	1.29 ± 0.07 ^d^
1281	Acetoin	1.17 ± 0.08 ^b^	2.29 ± 0.04 ^c^	2.34 ± 0.03 ^c^	2.85 ± 0.04 ^c^	2.34 ± 0.26 ^a^	2.81 ± 0.13 ^c^	3.18 ± 0.14 ^d^	3.44 ± 0.54 ^d,a^	2.60 ± 0.53 ^c^	2.68 ± 0.34 ^c^	3.06 ± 0.07 ^d^
1303	Acetol	0.85 ± 0.03 ^a^	0.20 ± 0.03 ^e^	0.34 ± 0.01 ^f^	0.74 ± 0.00 ^g^	0.90 ± 0.04 ^a^	0.99 ± 0.04 ^b^	1.09 ± 0.07 ^b^	1.04 ± 0.13 ^b^	0.47 ± 0.03 ^c^	0.57 ± 0.03 ^d^	1.37 ± 0.02 ^d^
	**Aldehydes**											
820	2-methylbutanal	9.56 ± 0.21 ^a^	10.69 ± 0.43 ^g^	11.49 ± 0.35 ^g^	8.84 ± 0.35 ^a^	8.79 ± 0.67 ^a^	3.25 ± 0.46 ^c^	3.67 ± 0.11 ^d^	7.13 ± 0.50 ^a^	2.87 ± 0.34 ^c^	7.30 ± 0.05 ^e^	7.75 ± 0.05 ^f^
825	3-methylbutanal	13.31 ± 2.41 ^a^	21.62 ± 0.01 ^c^	20.66 ± 0.19 ^c^	18.67 ± 0.76 ^d^	12.97 ± 2.17 ^a^	15.36 ± 0.48 ^a^	9.40 ± 0.24 ^b^	8.91 ± 0.67 ^b^	14.98 ± 2.11 ^a,c^	15.06 ± 2.11 ^a,c^	15.81 ± 0.19 ^a^
1078	Hexanal	6.91 ± 0.84 ^a^	12.90 ± 0.39 ^d^	9.83 ± 0.68 ^e^	8.40 ± 0.30 ^f^	6.41 ± 1.09 ^a^	4.72 ± 0.41 ^b^	3.81 ± 0.58 ^b^	2.84 ± 0.12 ^c^	4.88 ± 0.99 ^b^	3.85 ± 0.31 ^b^	3.08 ± 0.18 ^b^
1325	2-heptenal	0.19 ± 0.01 ^a^	0.57 ± 0.03 ^c^	0.73 ± 0.05 ^e^	0.95 ± 0.02 ^f^	0.20 ± 0.01 ^a^	1.14 ± 0.04 ^b^	1.11 ± 0.05 ^b^	1.12 ± 0.21 ^b^	0.56 ± 0.09 ^c^	0.85 ± 0.02 ^d^	1.12 ± 0.09 ^b^
1522	Benzaldehyde	1.92 ± 0.02 ^a^	5.03 ± 0.06 ^b^	7.15 ± 0.42 ^d^	7.65 ± 0.41 ^d^	1.72 ± 0.19 ^a^	4.07 ± 0.93 ^b^	4.90 ± 0.86 ^b^	5.87 ± 0.44 ^b^	3.75 ± 0.23 ^c^	4.34 ± 0.30 ^b^	5.20 ± 0.75 ^b^
1640	Benzeneacetaldehyde	Nd	2.10 ± 0.05 ^b^	2.40 ± 0.06 ^b^	3.12 ± 0.07 ^d^	Nd	1.76 ± 0.19 ^a^	2.32 ± 0.19 ^b^	2.45 ± 0.09 ^b^	1.78 ± 0.04 ^a^	1.85 ± 0.01 ^a^	6.65 ± 0.05 ^c^
	**Esters and acetates**											
1020	Ethyl butanoate	Nd	0.30 ± 0.01 ^d^	0.33 ± 0.02 ^d^	0.45 ± 0.03 ^c^	Nd	0.15 ± 0.01 ^a^	0.26 ± 0.02 ^b^	0.26 ± 0.01 ^b^	0.23 ± 0.02 ^b^	0.26 ± 0.01 ^b^	0.42 ± 0.04 ^c^
1229	Ethyl hexanoate	Nd	0.71 ± 0.03 ^c^	1.20 ± 0.03 ^d^	1.39 ± 0.05 ^b^	Nd	2.24 ± 0.13 ^a^	2.20 ± 0.14 ^a^	1.53 ± 0.24 ^b^	0.80 ± 0.14 ^c^	1.11 ± 0.20 ^d^	1.36 ± 0.01 ^b^
1433	Ethyl octanoate	0.14 ± 0.01 ^a^	0.72 ± 0.03 ^e^	0.78 ± 0.00 ^f^	0.82 ± 0.00 ^b^	Nd	0.87 ± 0.10 ^b^	0.82 ± 0.10 ^b^	0.92 ± 0.06 ^b^	0.67 ± 0.01 ^c^	0.60 ± 0.03 ^d^	0.68 ± 0.11 ^c^
801	Ethyl acetate	1.94 ± 0.04 ^b^	2.71 ± 0.03 ^f^	3.20 ± 0.05 ^h^	3.77 ± 0.09 ^i^	1.14 ± 0.01 ^a^	1.43 ± 0.09 ^c^	2.30 ± 0.15 ^d^	2.02 ± 0.31 ^d^	1.74 ± 0.06 ^a^	2.55 ± 0.15 ^d,f^	5.71 ± 1.23 ^g^
	**Alcohols**											
888	Ethanol	93.06 ± 0.10 ^b^	97.78 ± 4.10 ^a,b^	103.00 ± 0.69 ^f^	110.00 ± 1.66 ^c^	95.53 ± 0.23 ^a^	119.94 ± 6.55 ^c^	186.97 ± 2.70 ^d^	215.73 ± 4.86 ^a^	92.78 ± 4.10 ^a,b^	104.77 ± 0.69 ^f^	105.93 ± 1.66 ^f^
1211	Isoamylalcohol	26.73 ± 0.42 ^a^	24.17 ± 0.07 ^f^	27.96 ± 0.58 ^g^	34.36 ± 0.14 ^h^	28.97 ± 2.15 ^a^	17.48 ± 0.50 ^b^	20.23 ± 1.20 ^c^	32.55 ± 0.51 ^d^	12.60 ± 0.60 ^e^	16.90 ± 0.15 ^b^	24.46 ± 0.50 ^f^
1108	Isobutanol	3.25 ± 0.15 ^a^	3.60 ± 0.50 ^a^	2.64 ± 0.75 ^b^	1.76 ± 0.04 ^c^	3.30 ± 0.48 ^a^	3.31 ± 0.99 ^a^	2.51 ± 0.16 ^b^	2.33 ± 0.29 ^b^	3.33 ± 0.60 ^a^	2.51 ± 0.06 ^b^	1.81 ± 0.03 ^c^
1355	1-Hexanol	3.97 ± 0.14 ^a^	5.10 ± 0.55 ^a^	8.64 ± 0.56 ^e^	12.13 ± 0.69 ^f^	4.32 ± 0.30 ^a^	6.70 ± 0.36 ^b^	4.64 ± 0.42 ^a^	3.61 ± 0.41 ^c^	4.74 ± 0.15 ^d^	8.60 ± 0.21 ^e^	9.43 ± 0.40 ^f^
1905	Benzeneethanol	0.92 ± 0.02 ^a^	1.23 ± 0.01 ^c^	1.14 ± 0.03 ^c^	1.02 ± 0.06 ^a^	0.92 ± 0.05 ^a^	3.35 ± 0.51 ^b^	3.12 ± 0.65 ^b^	2.34 ± 0.58 ^b^	1.30 ± 0.09 ^c^	3.27 ± 0.19 ^b^	1.30 ± 0.21 ^c^
	**Pyrazines**											
1268	Methylpyrazine	2.60 ± 0.74 ^a^	5.39 ± 0.03 ^b^	5.48 ± 0.07 ^b^	6.10 ± 0.07 ^b^	2.80 ± 0.50 ^a^	5.86 ± 0.52 ^b^	7.79 ± 1.55 ^c^	12.89 ± 1.51 ^d^	5.26 ± 0.35 ^b^	5.87 ± 0.35 ^b^	9.63 ± 0.60 ^c^
1325	2,5-dimethyl pyrazine	0.75 ± 0.11 ^a^	1.42 ± 0.04 ^d^	1.50 ± 0.01 ^d^	1.67 ± 0.00 ^e^	0.80 ± 0.11 ^a^	1.19 ± 0.10 ^b^	1.83 ± 0.47 ^c^	1.91 ± 0.12 ^c^	1.10 ± 0.03 ^b^	1.25 ± 0.02 ^b^	1.47 ± 0.29 ^d^
1349	2.3-dimethylpyrazine	Nd	0.80 ± 0.04 ^c^	1.20 ± 0.06 ^f^	1.98 ± 0.05 ^g^	Nd	0.35 ± 0.01 ^a^	0.42 ± 0.03 ^b^	0.86 ± 0.05 ^c^	0.74 ± 0.01 ^d^	0.85 ± 0.05 ^c^	1.74 ± 0.01 ^e^
1386	2-ethyl-6-methylpyrazine	1.12 ± 0.10 ^b^	0.90 ± 0.02 ^g^	1.20 ± 0.04 ^b^	1.84 ± 0.00 ^d^	0.10 ± 0.01 ^a^	1.15 ± 0.04 ^b^	1.38 ± 0.04 ^c^	1.83 ± 0.04 ^d^	0.97 ± 0.02 ^e^	1.40 ± 0.04 ^c^	1.60 ± 0.02 ^f^
1391	2-ethyl-5-methylpyrazine	0.65 ± 0.02 ^b^	1.30 ± 0.05 ^f^	1.34 ± 0.10 ^g^	1.85 ± 0.20 ^e^	0.79 ± 0.02 ^a^	1.14 ± 0.07 ^c^	1.64 ± 0.12 ^d^	1.87 ± 0.13 ^e^	1.25 ± 0.01 ^f^	1.45 ± 0.03 ^g^	1.87 ± 0.02 ^e^
	**Acids**											
1447	Acetic acid	12.19 ± 0.30 ^b^	20.50 ± 0.40 ^c^	22.35 ± 0.55 ^c^	27.20 ± 0.43 ^e^	13.76 ± 0.79 ^a^	20.73 ± 0.72 ^c^	21.13 ± 0.75 ^c^	22.71 ± 2.00 ^c,d^	20.17 ± 3.66 ^c^	22.42 ± 1.01 ^c^	25.02 ± 1.53 ^d^
1628	Butanoic acid	0.64 ± 0.01 ^a^	0.43 ± 0.01 ^c^	0.55 ± 0.12 ^a^	0.67 ± 0.08 ^a^	0.59 ± 0.07 ^a^	1.03 ± 0.11 ^b^	0.98 ± 0.05 ^b^	1.03 ± 0.10 ^b^	0.44 ± 0.09 ^a,c^	0.45 ± 0.02 ^c^	0.70 ± 0.07 ^a^
1840	Hexanoic acid	0.20 ± 0.01 ^b^	1.12 ± 0.03 ^e^	1.23 ± 0.01 ^e^	1.40 ± 0.05 ^f^	0.27 ± 0.02 ^a^	0.70 ± 0.11 ^c^	0.90 ± 0.04 ^d^	1.19 ± 0.04 ^e^	1.07 ± 0.02 ^f^	1.08 ± 0.02 ^f^	1.17 ± 0.09 ^e^
2058	Octanoic acid	0.18 ± 0.00 ^a^	0.12 ± 0.00 ^c^	0.16 ± 0.06 ^c^	0.18 ± 0.06 ^c,a^	0.22 ± 0.04 ^a^	0.35 ± 0.02 ^b^	0.23 ± 0.02 ^a^	0.13 ± 0.01 ^c^	0.11 ± 0.02 ^c^	0.12 ± 0.00 ^c^	0.15 ± 0.01 ^c^
2281	Decanoic acid	0.30 ± 0.04 ^a^	0.34 ± 0.06 ^a^	0.40 ± 0.05 ^a^	0.48 ± 0.01 ^e^	0.35 ± 0.04 ^a^	0.25 ± 0.06 ^b^	0.56 ± 0.02 ^c^	0.81 ± 0.01 ^d^	0.30 ± 0.01 ^a^	0.25 ± 0.01 ^b^	0.28 ± 0.03 ^b^
	**Furans and pyrans**											
1223	2-pentylfuran	3.43 ± 0.26 ^a^	3.51 ± 0.05 ^g^	3.83 ± 0.05 ^d^	4.85 ± 0.68 ^e^	3.37 ± 0.35 ^a^	1.25 ± 0.04 ^b^	1.38 ± 0.03 ^c^	3.80 ± 0.09 ^d^	1.82 ± 0.02 ^e^	2.65 ± 0.10 ^f^	3.88 ± 0.6 ^d^
1462	2-furfural	4.22 ± 0.07 ^b^	6.10 ± 0.01 ^g^	5.48 ± 0.04 ^e^	4.25 ± 0.03 ^b^	3.80 ± 0.17 ^a^	10.37 ± 0.22 ^c^	8.61 ± 0.79 ^d^	4.25 ± 0.85 ^b^	5.42 ± 0.12 ^e^	3.95 ± 0.14 ^a,b^	3.05 ± 0.01 ^f^
1658	2-Furanmethanol	3.48 ± 0.39 ^a^	6.71 ± 0.06 ^e^	2.06 ± 0.08 ^f^	1.44 ± 0.08 ^g^	3.81 ± 0.55 ^a^	9.15 ± 0.07 ^c^	7.13 ± 0.54 ^d^	4.1 ± 0.56 ^a^	6.64 ± 0.11 ^d,e^	2.11 ± 0.28 ^f^	2.17 ± 0.25 ^f^
1503	2-acetylfuran	0.65 ± 0.05 ^b^	0.88 ± 0.02 ^i^	0.81 ± 0.00 ^a^	0.73 ± 0.02 ^a^	0.77 ± 0.06 ^a^	0.80 ± 0.03 ^a^	1.17 ± 0.22 ^d^	1.77 ± 0.10 ^e^	1.31 ± 0.02 ^f^	0.47 ± 0.01 ^g^	0.27 ± 0.01 ^h^
2501	5-(hydroxymethyl)-2-furfural (HMF)	0.35 ± 0.01 ^a^	0.30 ± 0.01 ^c^	0.35 ± 0.09 ^a^	0.46 ± 0.02 ^b^	0.31 ± 0.07 ^a^	0.46 ± 0.04 ^b^	0.48 ± 0.01 ^b^	0.39 ± 0.02 ^a^	0.27 ± 0.04 ^a^	0.24 ± 0.01 ^a^	0.28 ± 0.02 ^a^
	**Phenols**											
2203	Vinylguaiacol	Nd	0.71 ± 0.00 ^b^	0.76 ± 0.01 ^c^	0.81 ± 0.00 ^d^	Nd	0.61 ± 0.02 ^a^	0.63 ± 0.05 ^a^	0.69 ± 0.01 ^a, b^	0.70 ± 0.01 ^b^	0.75 ± 0.01 ^c^	0.79 ± 0.03 ^d^
1862	Guaiacol	Nd	0.60 ± 0.01 ^c^	0.75 ± 0.02 ^d^	0.88 ± 0.01 ^b^	Nd	0.46 ± 0.01 ^a^	0.87 ± 0.07 ^b^	0.99 ± 0.08 ^b^	0.52 ± 0.05 ^a^	0.63 ± 0.05 ^c^	0.72 ± 0.03 ^d^

RI: retention index. RIs were calculated using the van Den Dool and Kratz formula. Calculated RIs were compared using the online NIST database (http://webbook.nist.gov/chemistry/; accessed on 1 June 2023) for a high polar column for InnoWAX or similar stationary phases. All the compounds were identified by the matching RI and MS. The results are expressed as RAP ± SD; relative peak area (area peak compound/area peak internal standard) × 100 ± standard deviation. Values labeled with different lowercase letters in the same row are significantly different (*p* < 0.05). Nd: not detected.

**Table 9:** Eigenvalues of the Principal Component Analysis of the volatile organic compound analysis of soft wheat flour bread (SWB) samples. This table reports the eigenvalues of each component of the PCA as well as the percentage of the total variance that is accounted for by each component.

Axis	Eigenvalue	Difference	Proportion (%)	Cumulative (%)
1	17.114715	7.994542	40.75%	40.75%
2	9.120173	1.990407	21.71%	62.46%
3	7.129766	3.920977	16.98%	79.44%
4	3.208789	0.33098	7.64%	87.08%
5	2.877809	2.013748	6.85%	93.93%
6	0.864061	0.210451	2.06%	95.99%
7	0.65361	0.225627	1.56%	97.55%
8	0.427982	0.077306	1.02%	98.56%
9	0.350676	0.098257	0.83%	99.40%
10	0.25242	0.25242	0.60%	100.00%

**Table 10:** Eigenvalues of the Principal Component Analysis of the volatile organic compound analysis of whole wheat flour bread (WWB) samples. This table reports the eigenvalues of each component of the PCA as well as the percentage of the total variance that is accounted for by each component.

Axis	Eigen Value	Difference	Proportion (%)	Cumulative (%)
1	17.29995	6.696625	40.23%	40.23%
2	10.603324	5.611939	24.66%	64.89%
3	4.991385	0.937016	11.61%	76.50%
4	4.054369	1.117212	9.43%	85.93%
5	2.937157	1.670838	6.83%	92.76%
6	1.266318	0.531867	2.94%	95.70%
7	0.734452	0.244383	1.71%	97.41%
8	0.490069	0.152634	1.14%	98.55%
9	0.337434	0.051891	0.78%	99.34%
10	0.285543	0.285543	0.66%	100.00%

## Data Availability

The original contributions presented in the study are included in the article, further inquiries can be directed to the corresponding author.

## References

[1] Nunes M.A., Pimentel F.B., Costa A.S.G., Alves R.C., Oliveira M.B.P.P. (2016). Olive by-products for functional and food applications: Challenging opportunities to face environmental constraints. Innov. Food Sci. Emerg. Technol..

[2] Tura D., Gigliotti C., Pedò S., Failla O., Bassi D., Serraiocco A. (2007). Influence of cultivar and site of cultivation on levels of lipophilic and hydrophilic antioxidants in virgin olive oils (*Olea europea* L.) and correlations with oxidative stability. Sci. Hortic..

[3] Batuecas E., Tommasi T., Battista F., Negro V., Sonetti G., Viotti P., Fino D., Mancini G. (2019). Life Cycle Assessment of waste disposal from olive oil production: Anaerobic digestion and conventional disposal on soil. J. Environ. Manag..

[4] Carmona I., Aguirre I., Griffith D.M., García-Borrego A. (2023). Towards a circular economy in virgin olive oil production: Valorization of the olive mill waste (OMW) “alpeorujo” through polyphenol recovery with natural deep eutectic solvents (NADESs) and vermicomposting. Sci. Total Environ..

[5] Goula A.M., Lazarides H.N. (2015). Integrated processes can turn industrial food waste into valuable food by-products and/or ingredients: The cases of olive mill and pomegranate wastes. J. Food Eng..

[6] Harrami M., El Fami N., Moussadik A., Khachani N., Taibi M., Diouri A. (2022). Elaboration and characterization of composite clays based on “Coal waste-Olive pomace” mixtures. Mat. Today-Proc..

[7] Lila K., Belaadi S., Solimando R., Ralida Zirour F. (2020). Valorisation of organic waste: Use of olive kernels and pomace for cement manufacture. J. Clean. Prod..

[8] Ruschioni S., Loreto N., Foligni R., Mannozzi C., Raffaelli N., Zamporlini F., Pasquini M., Roncolini A., Cardinali F., Osimani A. (2020). Addition of olive pomace to feeding substrate affects growth performance and nutritional value of mealworm (*Tenebrio molitor* L.) larvae. Foods.

[9] Roselló-Soto E., Koubaa M., Moubarik A., Lopes R.P., Saraiva J.A., Boussetta N., Grimi N., Barba F.J. (2015). Emerging opportunities for the effective valorization of wastes and by-products generated during olive oil production process: Non-conventional methods for the recovery of high-added value compounds. Trend Food Sci. Technol..

[10] Ying D., Hlaing M.M., Lerisson J., Pitts K., Cheng L., Sanguansri L., Augustin M.A. (2017). Physical properties and FTIR analysis of rice-oat flour and maize-oat flour based extruded food products containing olive pomace. Food Res. Int..

[11] Balli D., Cecchi L., Innocenti M., Bellumori M., Mulinacci N. (2021). Food by-products valorisation: Grape pomace and olive pomace (pâté) as sources of phenolic compounds and fiber for enrichment of tagliatelle pasta. Food Chem..

[12] Simonato B., Trevisan S., Tolve R., Favati F., Pasini G. (2019). Pasta fortification with olive pomace: Effects on the technological characteristics and nutritional properties. LWT.

[13] Ribeiro T.B., Bonifácio-Lopes T., Morais P., Miranda A., Nunes J., Vicente A.A., Pintado M. (2021). Incorporation of olive pomace ingredients into yoghurts as a source of fibre and hydroxytyrosol: Antioxidant activity and stability throughout gastrointestinal digestion. J. Food Eng..

[14] Cecchi L., Schuster N., Flynn D., Bechtel R., Bellumori M., Innocenti M., Mulinacci N., Guinard J.X. (2019). Sensory profiling and consumer acceptance of pasta, bread, and granola bar fortified with dried olive pomace (pâté): A byproduct from virgin olive oil production. J. Food Sci..

[15] Cedola A., Cardinali A., D’Antuono I., Conte A., Del Nobile M.A. (2020). Cereal foods fortified with by-products from the olive oil industry. Food Biosci..

[16] Di Nunzio M., Picone G., Pasini F., Chiarello E., Caboni M.F., Capozzi F., Gianotti A., Bordoni A. (2020). Olive oil by-product as functional ingredient in bakery products. Influence of processing and evaluation of biological effects. Food Res. Int..

[17] Durante M., Bleve G., Selvaggini R., Veneziani G., Servili M., Mita G. (2019). Bioactive compounds and stability of a typical Italian bakery products “*Taralli*” enriched with fermented olive paste. Molecules.

[18] Miranda-Ramos K.C., Sanz-Ponce N., Haros C.M. (2019). Evaluation of technological and nutritional quality of bread enriched with amaranth flour. LWT.

[19] Taccari M., Aquilanti L., Polverigiani S., Osimani A., Garofalo C., Milanović V., Clementi F. (2016). Microbial diversity of type I sourdoughs prepared and back-slopped with wholemeal and refined soft (*Triticum aestivum*) wheat flours. J. Food Sci..

[20] Saka M., Özkaya B., Saka İ. (2021). The effect of bread-making methods on functional and quality characteristics of oat bran blended bread. Int. J. Gastr. Food Sci..

[21] Venturi M., Cappelli A., Pini N., Galli V., Lupori L., Granchi L., Cini E. (2022). Effects of kneading machine type and total element revolutions on dough rheoLogy and bread characteristics: A focus on straight dough and indirect (biga) methods. LWT.

[22] Dall’Asta C., Cirlini M., Morini E., Rinaldi M., Ganino T., Chiavaro E. (2013). Effect of chestnut flour supplementation on physico-chemical properties and volatiles in bread making. LWT.

[23] Martin-Diana A.B., Izquierdo N., Albertos I., Sanchez M.S., Herrero A., Sanz M.A., Rico D. (2017). Valorization of Carob’s Germ and Seed Peel as Natural Antioxidant Ingredients in Gluten-Free Crackers. J. Food Process. Preserv..

[24] Jemai H., Bouaziz M., Sayadi S. (2009). Phenolic composition, sugar contents and antioxidant activity of Tunisian sweet olive cultivar with regard to fruit ripening. J. Agric. Food Chem..

[25] Pulido R., Bravo L., Saura-Calixto F. (2000). Antioxidant activity of dietary polyphenols as determined by a modified ferric reducing/antioxidant power assay. J. Agric. Food Chem..

[26] Osimani A., Belleggia L., Botta C., Ferrocino I., Milanović V., Cardinali F., Haouet M.N., Garofalo C., Mozzon M., Foligni R. (2023). Journey to the morpho-textural traits, microbiota, and volatilome of Ciauscolo PGI salami. Food Biosci..

[27] Dreher J., Konig M., Herrmann K., Terjung N., Gibis M., Weiss J. (2021). Varying the amount of solid fat in animal fat mimetics for plant-based salami analogues influences texture, appearance and sensory characteristics. LWT.

[28] Cardinali F., Garofalo C., Reale A., Boscaino F., Osimani A., Milanovic V., Taccari M., Aquilanti L. (2022). Liquid sourdough from stone-ground soft wheat (*Triticum aestivum*) flour: Development and exploitation in the breadmaking process. Food Res. Int..

[29] Van Den Dool H., Kratz P.D. (1963). A Generalization of the retention index system including linear temperature programmed gas-liquid partition chromatography. J. Chromatogr. A.

[30] Foti P., Russo N., Randazzo C.L., Choupina A.B., Pino A., Caggia C., Romeo F.V. (2023). Profiling of phenol content and microbial community dynamics during pâté olive cake fermentation. Food Biosci..

[31] Zhou D., Zhong J., Huang Y., Cheng Y. (2023). Effect of free and bound polyphenols from Rosa roxburghii Tratt distiller’s grains on moderating fecal microbiota. Food Chem. X.

[32] Piscopo A., De Bruno A., Zappia A., Poiana M. (2014). Antioxidant activity of dried green olives (Carolea cv.). LWT.

[33] Pellegrini N., Serafini M., Colombi B., Del Rio D., Salvatore S., Bianchi M., Brighenti F. (2003). Total antioxidant capacity of plant foods, beverages and oils consumed in Italy assessed by three different in vitro assays. J. Nutr..

[34] Marinopoulou A., Papageorgiou M., Irakli M., Gerasopoulos D. (2020). Effect of Olive Pulp Enrichment on Physicochemical and Antioxidant Properties of Wheat Bread. Int. J. Food Stud..

[35] Peng X., Ma J., Cheng K.W., Jiang Y., Chen F., Wang M. (2010). The effects of grape seed extract fortification on the antioxidant activity and quality attributes of bread. Food Chem..

[36] Batista A.P., Niccolai A., Bursic I., Sousa I., Raymundo A., Rodolfi L., Biondi N., Tredici M.R. (2019). Microalgae as Functional Ingredients in Savory Food Products: Application to Wheat Crackers. Foods.

[37] EFSA Panel on Dietetic Products, Nutrition, and Allergies (NDA) (2010). Scientific opinion on dietary reference values for carbohydrates and dietary fibre. EFSA J..

[38] Pereira A.P.M., Stradiotto G.C., Freire L., Alvarenga V.O., Crucello A., Morassi L.L.P., Silva F.P., Sant’Ana A.S. (2020). Occurrence and enumeration of rope-producing spore forming bacteria in flour and their spoilage potential in different bread formulations. LWT.

[39] O’Shea N., Kilcawley K.N., Gallagher E. (2017). Aromatic composition and physicochemical characteristics of crackers containing barley fractions. Cereal Chem..

[40] de Gennaro G., Difonzo G., Summo C., Pasqualone A., Caponio F. (2022). Olive cake powder as functional ingredient to improve the quality of gluten-free breadsticks. Foods.

[41] Pasqualone A., Bianco A.M., Paradiso V.M., Summo C., Gambacorta G., Caponio F., Blando A. (2015). Production and characterization of functional biscuits obtained from purple wheat. Food Chem..

[42] Choe E., Min D.B. (2006). Mechanisms and factors for edible oil oxidation. Compr. Rev. Food Sci. Food Saf..

